# Rhizobia–diatom symbiosis fixes missing nitrogen in the ocean

**DOI:** 10.1038/s41586-024-07495-w

**Published:** 2024-05-09

**Authors:** Bernhard Tschitschko, Mertcan Esti, Miriam Philippi, Abiel T. Kidane, Sten Littmann, Katharina Kitzinger, Daan R. Speth, Shengjie Li, Alexandra Kraberg, Daniela Tienken, Hannah K. Marchant, Boran Kartal, Jana Milucka, Wiebke Mohr, Marcel M. M. Kuypers

**Affiliations:** 1 Max Planck Institute for Marine Microbiology, Bremen, Germany; 2 Centre for Microbiology and Environmental Systems Science, Division of Microbial Ecology, University of Vienna, Vienna, Austria; 3 Alfred Wegener Institute, Helmholtz Centre for Polar and Marine Research, Bremerhaven, Germany; 4 MARUM – Centre for Marine Environmental Sciences, University of Bremen, Bremen, Germany; 5 School of Science, Constructor University, Bremen, Germany; 6 Present Address: Department of Microbiology, University of Innsbruck, Innsbruck, Austria; 7 Present Address: Alfred Wegener Institute, Helmholtz Centre for Polar and Marine Research, Bremerhaven, Germany; 8 Present Address: Centre for Microbiology and Environmental Systems Science, Division of Microbial Ecology, University of Vienna, Vienna, Austria

**Keywords:** Element cycles, Ocean sciences, Biogeochemistry

## Abstract

Nitrogen (N_2_) fixation in oligotrophic surface waters is the main source of new nitrogen to the ocean^1^ and has a key role in fuelling the biological carbon pump^2^. Oceanic N_2_ fixation has been attributed almost exclusively to cyanobacteria, even though genes encoding nitrogenase, the enzyme that fixes N_2_ into ammonia, are widespread among marine bacteria and archaea^3–5^. Little is known about these non-cyanobacterial N_2_ fixers, and direct proof that they can fix nitrogen in the ocean has so far been lacking. Here we report the discovery of a non-cyanobacterial N_2_-fixing symbiont, ‘*Candidatus* Tectiglobus diatomicola’, which provides its diatom host with fixed nitrogen in return for photosynthetic carbon. The N_2_-fixing symbiont belongs to the order Rhizobiales and its association with a unicellular diatom expands the known hosts for this order beyond the well-known N_2_-fixing rhizobia–legume symbioses on land^6^. Our results show that the rhizobia–diatom symbioses can contribute as much fixed nitrogen as can cyanobacterial N_2_ fixers in the tropical North Atlantic, and that they might be responsible for N_2_ fixation in the vast regions of the ocean in which cyanobacteria are too rare to account for the measured rates.

## Abstract (web summary)

A symbiosis between a diatom and a newly discovered species of alphaproteobacteria, ‘*Candidatus* Tectiglobus diatomicola’, can fix nitrogen in the ocean, providing evidence that nitrogen fixers other than cyanobacteria have a key role in the marine environment.

## Main

Nitrogen is an essential component of all living organisms and limits life in the ocean. Atmospheric N_2_ gas is the largest reservoir of freely accessible nitrogen, but it is biologically available only to microorganisms that carry the nitrogenase metalloenzyme and thus can fix N_2_ into ammonia^7^. Even though a wide diversity of marine bacteria and archaea encode nitrogenase, the bulk of nitrogen fixation in the ocean has been attributed to cyanobacteria (ref. ^4^ and references therein). These phototrophs are capable of both free-living and symbiotic lifestyles, and can directly or indirectly contribute to carbon fixation and export production in the regions where they are abundant, such as oligotrophic coastal waters and margins of subtropical gyres^8^. Notably, in vast regions of the ocean, such as the centres of subtropical gyres, cyanobacterial N_2_ fixers are too rare to account for the measured rates of N_2_ fixation. Instead, a role of non-cyanobacterial N_2_ fixers has been invoked, on the basis of the abundance of nitrogenase-encoding gene sequences (*nifH*), most of which belong to uncultured proteobacteria (for example, refs. ^3,5,9–11^). So far, the most frequently detected non-cyanobacterial N_2_ fixer is the so-called gamma-A, named after its *nifH* gene phylogeny that clusters within the Gammaproteobacteria^12^. This enigmatic microorganism has been shown to be distributed in most world oceans, and its potential activity has been inferred from in situ *nifH* transcription^13,14^. To date, however, there is no proof that gamma-A fixes N_2_ in situ, and essentially all aspects of its physiology remain unknown.

## An N_2_-fixing rhizobial diatom endophyte

**Fig. 1:**
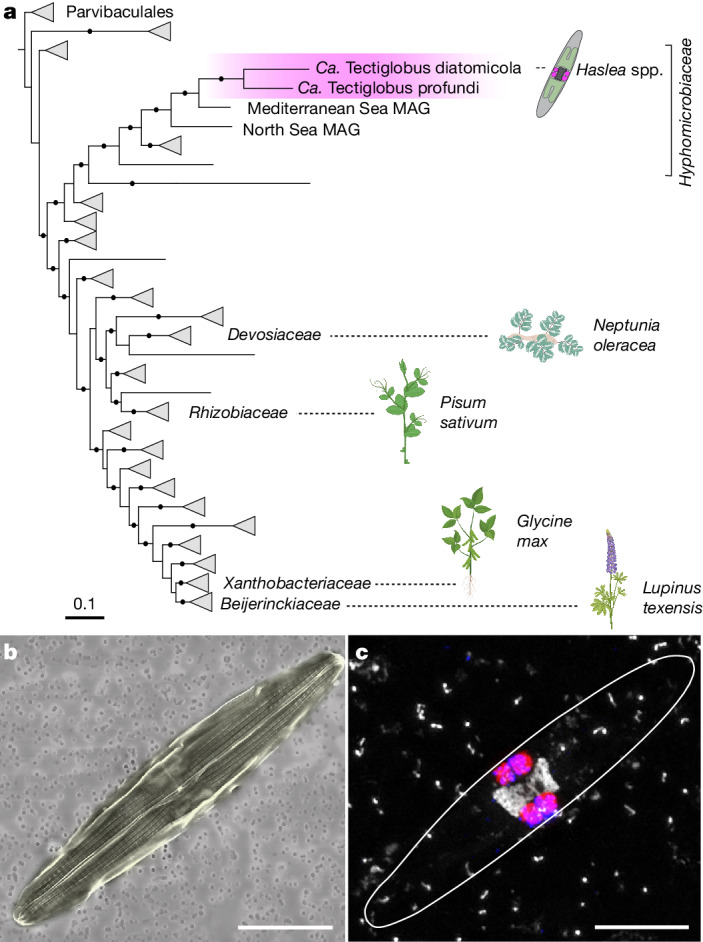
Phylogeny and visualization of *Candidatus* Tectiglobus diatomicola and its diatom host. **a**, Maximum likelihood phylogenetic tree of concatenated bacterial marker genes from the order Rhizobiales, showing the placement of *Ca*. T. diatomicola within the *Hyphomicrobiaceae* family (see Methods). The novel genus *Ca*. Tectiglobus, comprising *Ca*. T. diatomicola and its closest relative *Ca*. T. profundi, is highlighted in pink. Families within the Rhizobiales that contain known N_2_-fixing legume symbionts and their exemplary host plants are shown. The order Parvibaculales was used as an outgroup. Black dots indicate more than 95% bootstrap support. Scale bar indicates amino acid substitutions per site. Plant icons were designed by Freepik (*Neptunia oleracea*) or created with BioRender.com. **b**,**c**, False coloured scanning electron microscopy (SEM) image (**b**) and confocal laser scanning microscopy image (**c**) of a *Haslea* diatom. Four *Ca*. T. diatomicola cells (pink, overlay of Hypho1147 and Hypho734 fluorescence in situ hybridization (FISH) probes; Extended Data Table 2) were detected next to the host nucleus (white; stained with DAPI). Scale bars, 5 µm.

**Extended Data Fig. 1:**
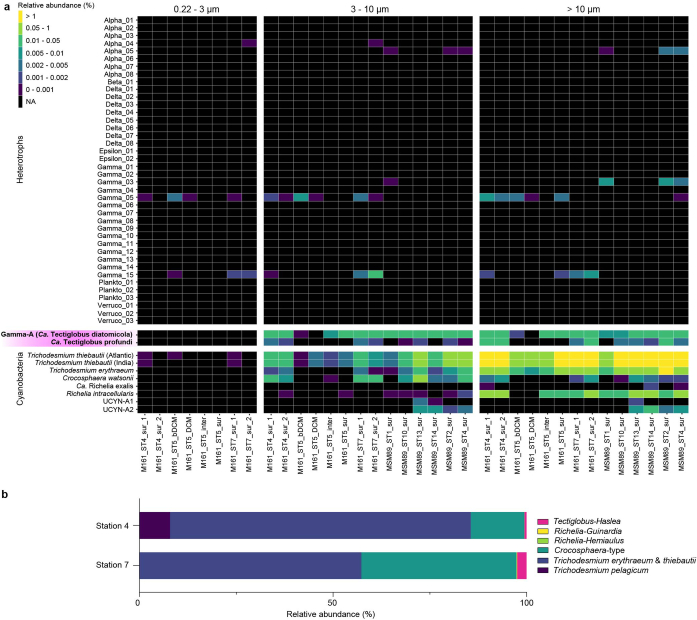
Relative abundance of heterotrophic and cyanobacterial N_2_ fixers in the tropical North Atlantic. **a**, Relative abundance of ‘*Ca*. T. diatomicola’ (gamma-A), ‘*Ca*. T. profundi’, as well as selected cosmopolitan heterotrophic and cyanobacterial N_2_ fixers^5^ based on read detection in metagenome data from different size fractions from the tropical North Atlantic. Note that only few known heterotrophic N_2_ fixers were detected in the dataset, and that ‘*Ca*. T. diatomicola’ had the highest relative abundance. **b**, Relative abundance of the most abundant known N_2_ fixers determined from direct microscopy counts (surface waters of stations 4 and 7 from cruise M161 in the tropical North Atlantic). Note that the metagenome based relative abundances of *Trichodesmium* in the large size fraction overestimate its microscopy-based abundance by an order of magnitude, most likely due to the polyploidy of this genus^94^. By contrast, metagenome based relative abundances of *Crocosphaera* in the large size fraction underestimate its microscopy-based abundance, most likely due to DNA extraction biases^95^. Source Data

**Extended Data Fig. 2:**
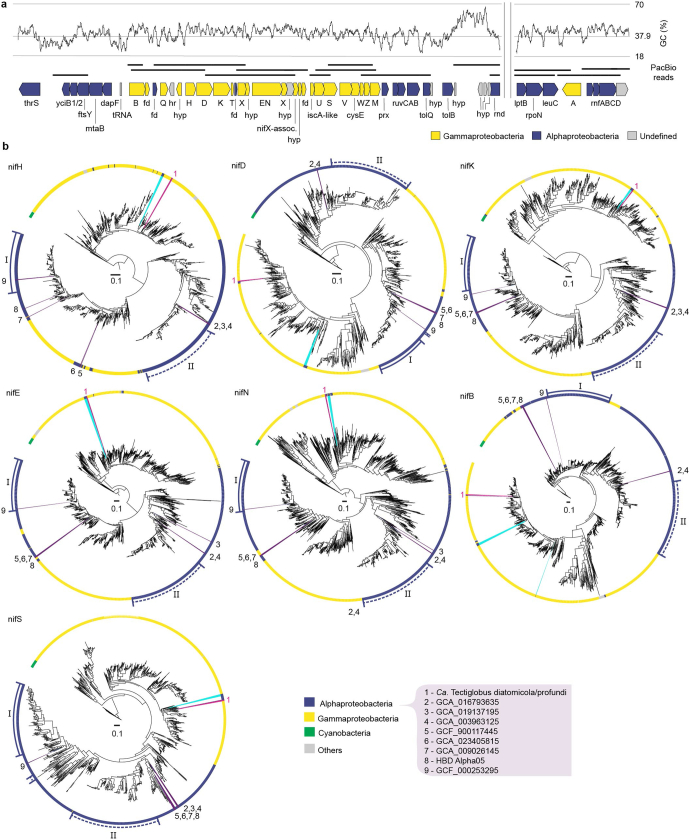
Organization and phylogeny of nitrogen fixation (*nif*) genes in ‘*Candidatus* Tectiglobus diatomicola’. **a**, Organization of the *nif* regulon showing the phylogenetic affiliation and GC content of each gene. All *nif* genes are affiliated to gammaproteobacteria (yellow), whereas almost all other genes in and around the *nif* regulon are affiliated to alphaproteobacteria (blue). The presence of *nifV* indicates that unlike most nodulating rhizobia, ‘*Ca*. T. diatomicola’ has the capacity to synthesize homocitrate, a ligand of the FeMo cofactor^6^. Genes affiliated to gamma-and alphaproteobacteria were found together on individual PacBio reads (solid black lines). Note that there is no substantial difference in GC content between genes of gamma- and alphaproteobacterial origin. **b**, Maximum likelihood phylogenetic trees of NifHDKENBS sequences of the *Pseudomonadota*. Phylogeny is based on the alignment of full-length amino acid sequences retrieved from the GTDB. Cyanobacterial Nif sequences were used as outgroups. All ‘*Ca*. Tectiglobus diatomicola’/‘*Ca*. Tectiglobus profundi’ (pink line; 1) Nif protein sequences cluster with gammaproteobacterial sequences, while Nif sequences from other members of the *Hyphomicrobiaceae* family (purple lines; 2–9; Extended Data Fig. 3) cluster with alphaproteobacteria. These alphaproteobacterial Nif protein sequences form deeply branching sister clades to two of the major nodulating Rhizobiales *nif* clusters; i.e. *Bradyrhizobium* (I, solid line) and *Allorhizobium-Mesorhizobium-Rhizobium-Sinorhizobium* (II, dashed line). Note that some other *Rhizobiales* (turquoise lines; members of the *Rhodobiaceae*, *Rhizobiaceae*, *Cohaesibacteraceae* and BM303 families) Nif protein sequences also cluster with the gammaproteobacterial sequences indicating that they were also obtained via horizontal gene transfer from a (common) gammaproteobacterial donor. Tree scales indicate amino acid substitutions per site. Note that in panel A, individual *nif* genes are indicated by capital letters; fd, ferredoxin; hr, hemerythrin; hyp, hypothetical protein; prx, peroxiredoxin; rnd, ribonuclease D.

We investigated the role of non-cyanobacterial N_2_ fixation in the tropical North Atlantic during an expedition in January–February 2020. This region is responsible for around 20% of oceanic N_2_ fixation^8^, and cyanobacteria can only explain approximately half of the rates measured in the region^10^. We detected high N_2_ fixation rates of up to 40 nmol N l^−1^ d^−1^ in the surface waters (Extended Data Table 1), and the presence of both cyanobacterial and heterotrophic N_2_ fixers—specifically, gamma-A—was confirmed by metagenomic sequencing (Extended Data Fig. 1a). Gamma-A *nifH* sequences were retrieved only from the large size fraction (greater than 3 µm) suggesting particle attachment or an association with a host organism (Extended Data Fig. 1a). We recovered a near-complete metagenome-assembled genome (MAG; 1.7 Mb, 37.8% GC, 98% completion with 0% redundancy) containing the gamma-A *nifH* gene, as well as a complete cluster of rRNA genes (Supplementary Table 1). Although the retrieved *nifH* sequence clustered within the Gammaproteobacteria as previously reported^3,14,15^ (Extended Data Fig. 2), both 16S-rRNA-gene-based and whole-genome-based taxonomy^16^ firmly placed this MAG within the alphaproteobacterial family *Hyphomicrobiaceae* (Fig. 1a). This family belongs to the order Rhizobiales, which comprises the prominent rhizobial symbionts of nodule-forming terrestrial legumes^6,17,18^. In addition to *nifH*, most other genes of the *nif* regulon are of gammaproteobacterial origin, including *nifD* and *nifK*, which encode the catalytic component of the nitrogenase; *nifE*, *nifN* and *nifB*, which encode the iron-molybdenum cofactor assembly proteins; and *nifS*, which is involved in metallocluster biosynthesis (Extended Data Fig. 2a). Almost all other genes in the gamma-A MAG are of alphaproteobacterial origin (Supplementary Table 1). On the basis of these results, we conclude that the gamma-A N_2_ fixer is, in fact, an alphaproteobacterium that has acquired its nitrogenase genes through horizontal gene transfer from a gammaproteobacterial donor. Besides gamma-A, several other bacteria, including members of the order Rhizobiales, obtained their nitrogenase genes through horizontal gene transfer from a gammaproteobacterial donor (Extended Data Fig. 2b). Such horizontal gene transfer across classes, resulting in the acquisition of nitrogenase genes, has been reported previously for other N_2_ fixers^19,20^.

**Extended Data Fig. 3:**
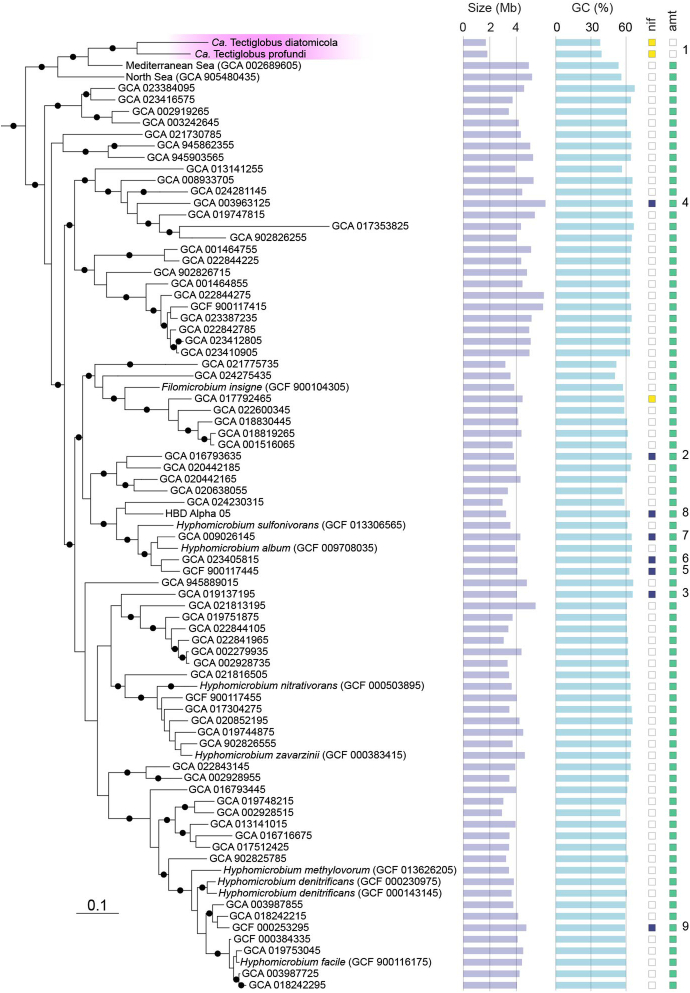
Maximum likelihood phylogenetic tree of concatenated bacterial marker genes, highlighting genomic features of the *Hyphomicrobiaceae* family. Columns from left to right represent genome size (Mb), GC content (%), presence and phylogeny of *nifH* genes (yellow, gammaproteobacteria; blue, alphaproteobacteria), and presence (green) of ammonium transporter genes (*amt*). The numbers on the right indicate the *nif*-containing members of the *Hyphomicrobiaceae* from Extended Data Fig. 2. ‘*Ca*. T. diatomicola’ and ‘*Ca*. T. profundi’ are highlighted in pink. Only genomes with >80% completion are shown. Black dots indicate >95% bootstrap support. Scale bar indicates amino acid substitutions per site. Source Data

We name the newly discovered species ‘*Candidatus* Tectiglobus diatomicola’ within a novel genus ‘*Candidatus* Tectiglobus’ (see Methods for etymology). One other marine MAG from the North Pacific, which we now name ‘*Candidatus* Tectiglobus profundi’, is affiliated with this novel genus, with 72% average amino acid identity with *Ca*. T. diatomicola (Supplementary Methods). Compared with their closest relative, a MAG from the Mediterranean Sea, both *Ca*. Tectiglobus species have a substantially reduced genome size (around 1.7 Mb versus around 5 Mb) and a strongly decreased GC content (around 38% versus around 54%) (Extended Data Fig. 3), which are features typical of endosymbionts^21^. Notably, a similar reduction in genome size and GC content is observed for the N_2_-fixing cyanobacterial endosymbiont *Candidatus* Atelocyanobacterium thalassa, or UCYN-A, which lives in symbiosis with a haptophyte alga^22,23^. Thus, the genome properties of *Ca*. T. diatomicola, together with its presence in the large size fraction, strongly indicate a host-associated lifestyle.

**Extended Data Fig. 4:**
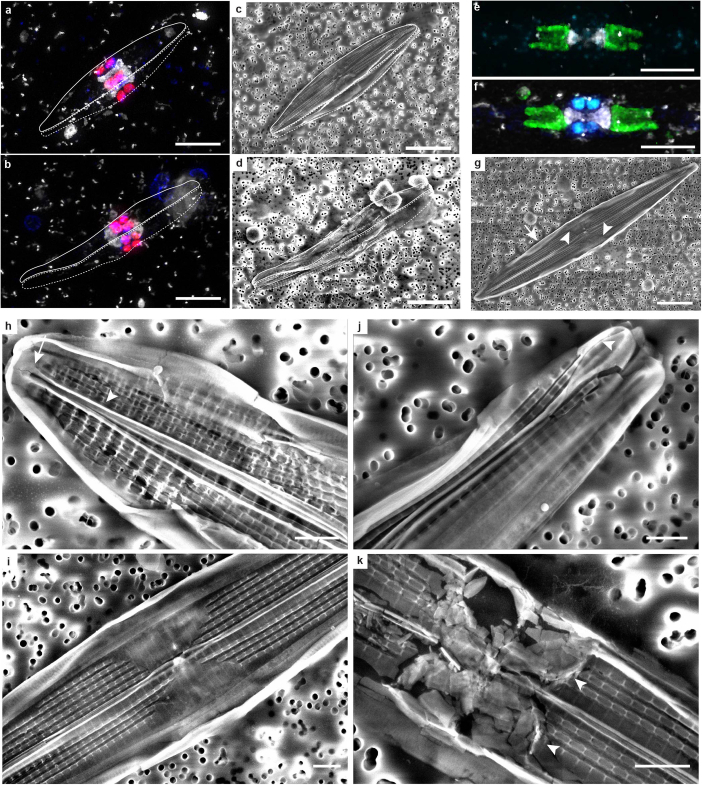
Microscopic characterization of ‘*Candidatus* Tectiglobus diatomicola’–*Haslea* symbioses. **a**–**d**, Confocal laser scanning microscopy images (**a**,**b**) and corresponding scanning electron micrographs (**c**,**d**) of dividing diatoms with six (**a**,**c**) and eight (**b**,**d**) ‘*Ca*. T. diatomicola’ cells, after hybridization with oligonucleotide probes (in pink, overlay of Hypho1147 and Hypho734 in red and blue, respectively; see Extended Data Table 2) and counterstaining with DAPI (white). Solid and dashed lines indicate assumed outline of the dividing diatoms. **e**, Confocal laser scanning microscopy image of a *Haslea* diatom after CARD-FISH with the NON338-probe showing no unspecific binding at the typical symbiont location (autofluorescence of the chloroplasts at 488 nm in green, NON338-probe background signal in light blue, DAPI in white). **f**, Epifluorescence image of the ‘*Ca*. T. diatomicola’–*Haslea* symbiosis, showing ‘*Ca*. T. diatomicola’ cells after hybridization with a specific oligonucleotide probe (Hypho638 in blue), autofluorescence of the chloroplasts in green and the H-shaped nucleus counterstained with DAPI (white). The bilobed chloroplasts are located on either side of the central valve area, and the lobes of the chloroplasts are connected along the cell’s transapical axis. **g**–**k**, Scanning electron micrographs of the diatom host. **g**, Whole frustule showing the internal raphe structure and an apparent hyaline area around the central raphe region (arrow) as well as sections of the hyaline girdle bands (arrowheads). **h**, Valve end showing the ridges bordering the central raphe. **i**, Central valve area. The valve’s normal surface structure of longitudinal ribs overlying the transapical striae appears modified with the longitudinal ribs damaged and only the transapical structures visible. **j**, Valve pole showing the shape of raphe end (arrow head). **k**, Central valve area with the central valve area collapsed. Arrowheads point to a chamber-like structure. Scale bars are 5 µm in **a**–**g** and 1 µm in **h**–**k**.

**Extended Data Fig. 5:**
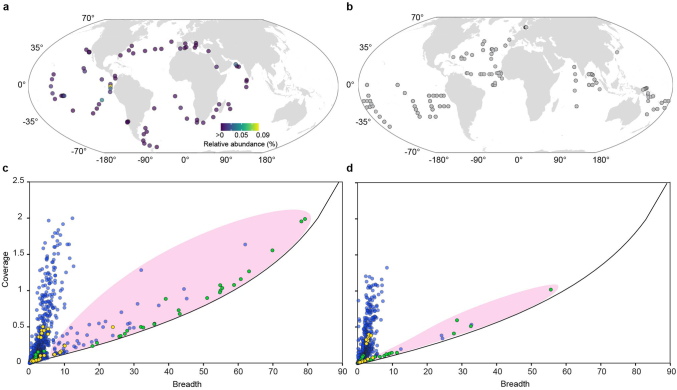
Detection of ‘*Candidatus* Tectiglobus’ and *Haslea* species in metagenomic and *nifH* qPCR datasets. **a**, Relative abundance of *Haslea* in the 0.8 to 5 µm size fraction determined from V9-18S rRNA amplicon sequencing data from Tara Oceans^96^. **b**, qPCR datasets in which no ‘*Ca*. T. diatomicola’ *nifH* were detected (grey circles; data from Shao et al. ^8,67^). **c**,**d**, Identification of ‘*Ca*. Tectiglobus’ in metagenomes from Tara Oceans (blue), the South Pacific gyre (yellow) and the tropical North Atlantic (green) datasets. Coverage and breadth (fraction of the genome covered by at least one read) of the ‘*Ca*. T. diatomicola’ (**c**) and ‘*Ca*. T. profundi’ (**d**) genomes within the metagenomic samples. The black line indicates the expected breadth, see Methods. ‘*Ca*. Tectiglobus’ was only considered to be present in metagenomes where the actual breadth was close to the expected breadth (all metagenomes inside the pink-shaded ellipses). Source Data

We designed specific 16S rRNA oligonucleotide probes to visualize *Ca*. T. diatomicola (Extended Data Table 2), and found hybridized cells (1–2 µm cocci) that were located exclusively inside diatom hosts (Fig. 1b,c and Extended Data Fig. 4). The hosts showed large variation in cell sizes (20–58 μm long and 3–8 μm wide), probably representing different diatom life stages^24^. Typically, four *Ca*. T. diatomicola symbionts were observed in the proximity of the centrally located host nucleus, with some dividing hosts containing up to eight symbionts (Fig. 1c and Extended Data Fig. 4). On the basis of scanning electron micrographs combined with fluorescence microscopy, the host was identified as a pennate diatom likely to belong to the genus *Haslea* within the *Naviculaceae* family (Extended Data Fig. 4; see also Supplementary Information). Indeed, sequences belonging to this genus were recovered from the metagenome with the highest abundance of *Ca*. T. diatomicola (Supplementary Table 2, see also Methods). *Haslea* are ubiquitous marine diatoms that are found in surface waters (Extended Data Fig. 5a) and coastal sediments throughout the world’s oceans^25^, but they have not previously been reported to contain N_2_-fixing symbionts. Most marine diatom species that form associations with N_2_-fixing cyanobacteria are centric diatoms such as *Hemiaulus*, *Rhizosolenia* and *Chaetoceros*^26^. The new *Haslea* hosts thus expand the range of diatoms that can associate with N_2_-fixing symbionts. More importantly, all so-far-known N_2_-fixing symbionts of diatoms belong exclusively to the cyanobacteria^27,28^. Our discovery represents the first example—to our knowledge—of a symbiosis between a diatom and a non-cyanobacterial N_2_-fixing microorganism.

## Host–symbiont metabolic interactions

**Fig. 2:**
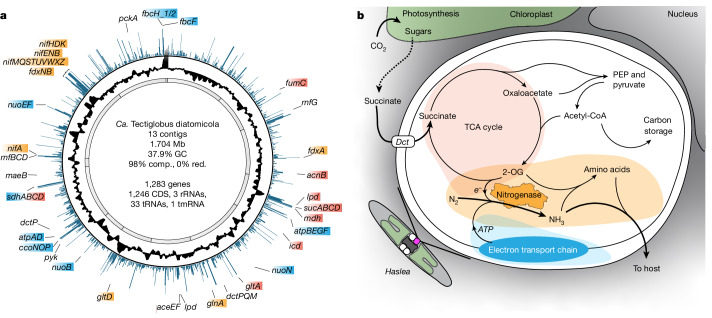
Genome properties, gene transcription and proposed metabolism of the *Candidatus* Tectiglobus diatomicola symbiont. **a**, Circular representation of the *Ca*. T. diatomicola genome with 13 encoding contigs (grey), GC content (black) and the average transcription of protein-coding genes as transcripts per million (TPM) (blue; TPM values higher than 800 were cut off). Genes related to N_2_ fixation (orange), electron transport chain and ATP generation (blue) and the TCA cycle (red) are highlighted. CDS, coding sequence; comp., completeness; red., redundancy; tmRNA, transfer-messenger RNA. **b**, Schematic of the proposed metabolic potential of *Ca*. T. diatomicola (white) and its interactions with *Haslea* (grey and green), indicating the transfer of fixed nitrogen from the N_2_-fixing symbiont in return for diatom-derived C_4_-dicarboxylic acids, such as succinate. Proteins and corresponding gene names are: Complex I (NADH–quinone oxidoreductase, *nuoBNEF*), Complex II (succinate dehydrogenase, *sdhABCD*), Complex III (cytochrome *b/c*1, *fbcH_1/2*, *fbcF*), Complex IV (*cbb*_*3*_-type oxidase, *ccoNOP*), Complex V (ATP synthase, *atpABDEGF*); fumarate hydratase (*fumC*); aconitate hydratase (*acnB*); 2-oxoglutarate dehydrogenase (*sucAB*, *lpd*); succinyl-CoA synthetase (*sucCD*); malate dehydrogenase (*mdh*); isocitrate dehydrogenase (*icd*); citrate synthase (*gltA*); nitrogenase (*nifHDK*) and its ancillary proteins (*nifAENBMQSTUVWXZ*) and ferredoxins (*fdxABN*); rnf complex (*rnfBCD*); dicarboxylic acid transporter (*dctPQM*); pyruvate dehydrogenase (*aceEF*, *lpd*); pyruvate kinase (*pyk*); malic enzyme (*maeB*); and phosphoenolpyruvate carboxykinase (*pckA*). 2-OG, 2-oxoglutarate; PEP, phosphoenolpyruvate.

To gain insights into the metabolic interactions between *Ca*. T. diatomicola and its *Haslea* host, we studied the *Ca*. T. diatomicola genome together with its in situ transcriptome. The *Ca*. T. diatomicola genome encodes all genes necessary for N_2_ fixation to ammonia, most of which were highly transcribed (Fig. 2a,b and Supplementary Table 1). These include key genes encoding the nitrogenase (*nifH*, *nifD* and *nifK*) and the iron-molybdenum cofactor assembly proteins (*nifE*, *nifN* and *nifB*).

*Ca*. T. diatomicola has a strongly reduced genome size, but the genome still encodes core carbon-processing pathways such as glycolysis and the tricarboxylic acid (TCA) cycle, which are present in many heterotrophic bacteria. However, on the basis of the low transcription of glycolysis genes (Supplementary Table 1), *Ca*. T. diatomicola probably does not grow on sugars. Instead, many genes involved in the TCA cycle were highly transcribed, in particular malate (*mdh*) and succinate (*sdh*) using enzymes (Fig. 2a,b and Supplementary Table 1), indicating growth on dicarboxylic acids. The dicarboxylates can be converted via pyruvate to acetyl-CoA, driving the TCA cycle independent of the glycolysis pathway (Fig. 2b). This is supported by the high transcription of genes encoding a TRAP-type dicarboxylic acid transporter (*dctP*, *dctQ* and *dctM*) and enzymes that decarboxylate malate (*maeB*) and oxaloacetate (*pckA* and *pyk*) to phosphoenolpyruvate and pyruvate. On the basis of the combined genomic and transcriptomic data, it seems that the N_2_-fixing *Ca*. T. diatomicola provides ammonia to the *Haslea* diatom host in return for dicarboxylic acids (Fig. 2b). This metabolite exchange is strongly reminiscent of the metabolic interaction in rhizobia–legume symbioses^29,30^, in which N_2_-fixing rhizobia grow on host-provided dicarboxylic acids, such as succinate and malate, and in return provide fixed nitrogen to the host plant. By contrast, in symbioses between marine diatoms and N_2_-fixing cyanobacteria, both partners are photosynthetic and grow on inorganic carbon^31^.

**Extended Data Fig. 6:**
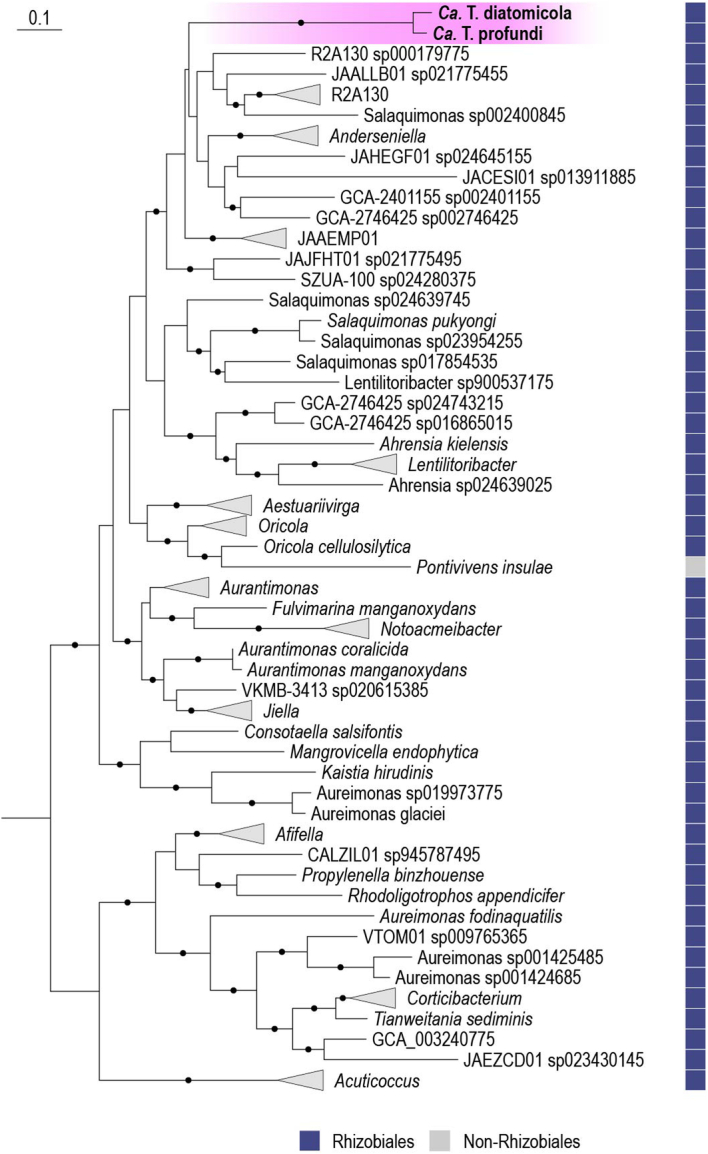
Maximum likelihood phylogenetic tree of the ‘*Candidatus* Tectiglobus diatomicola’ and ‘*Candidatus* Tectiglobus profundi’ high-affinity cytochrome *cbb*_3_-type terminal oxidase. The tree is constructed from closely related full-length CcoN amino acid sequences retrieved from the GTDB. The CcoN sequences of the two ‘*Ca*. Tectiglobus’ species are highlighted in pink. The coloured squares indicate the taxonomic affiliation of the genome. Black dots indicate > 95% bootstrap support. Scale bar indicates amino acid substitutions per site.

Notably, *Ca*. T. diatomicola seems to have lost its low-affinity terminal oxidase (Supplementary Table 1), which is typically present in other members of the *Hyphomicrobiaceae* family, with the notable exception of *Ca*. T. profundi (Supplementary Table 3). Instead, *Ca*. T. diatomicola encodes and highly transcribes the high-affinity cytochrome *cbb*_3_-type (*ccoN*, *ccoO* and *ccoP*) terminal oxidase (Fig. 2a, Extended Data Fig. 6 and Supplementary Table 1), which is used for respiration under low-oxygen conditions, and is generally poorly transcribed in high-oxygen environments such as the oxic surface waters of the tropical North Atlantic^32^. Legume-associated N_2_-fixing rhizobia also rely on high-affinity terminal oxidases when growing symbiotically^29^, because the plant hosts restrict the oxygen supply to the symbionts to control their growth and optimize nitrogen fixation^33^. The legume hosts also suppress the activity of the AMT-type ammonium transporters of nodulating rhizobia, to prevent the uptake of ammonium by the bacteria and to enhance ammonium transfer to the plant^30^. The lack of AMT transporters in *Ca*. T. diatomicola would similarly maximize the transfer of ammonia to the *Haslea* host. *Ca*. T. diatomicola seems to lack the capacity for de novo biosynthesis of some essential amino acids (aromatic amino acids, histidine and proline) and vitamins (for example, biotin and thiamine; Supplementary Table 1), a trait also found in nodulating Rhizobiales that are dependent on their plant host for these essential compounds^34,35^. Together, these results indicate that, similarly to nodulating rhizobia in legume symbioses, growth and N_2_ fixation by *Ca*. T. diatomicola is tightly regulated by its host.

**Fig. 3:**
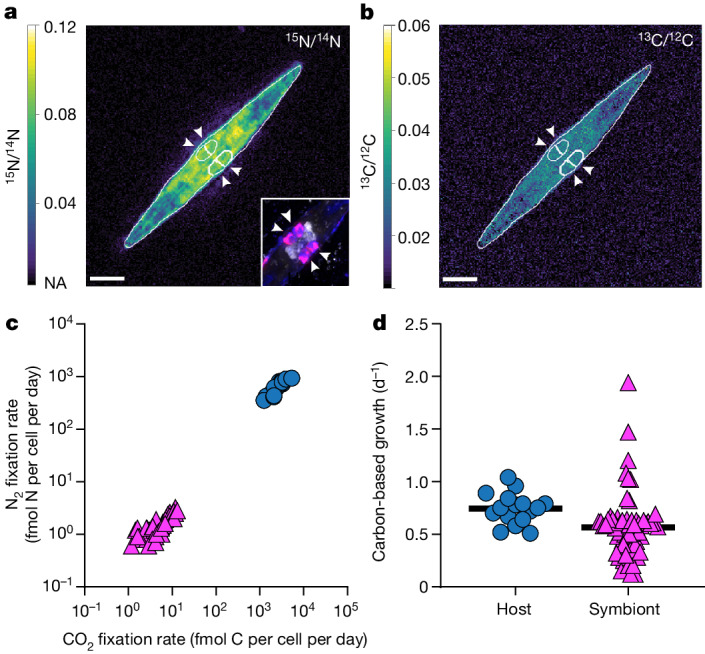
Activity of the *Candidatus* Tectiglobus diatomicola symbiont and its diatom host. **a**,**b**, NanoSIMS images showing the enrichment in ^15^N from ^15^N_2_ fixation (**a**) and ^13^C from ^13^CO_2_ fixation (**b**). The inset shows the corresponding fluorescence image after hybridization of *Ca*. T. diatomicola cells (indicated by white arrowheads) with specific oligonucleotide probes (in pink, overlay of Hypho638–Hypho825 mix in blue and Hypho1147 in red, respectively) (Extended Data Table 2). Scale bars, 5 µm. **c**, Cellular CO_2_ and N_2_ fixation rates of *Ca*. T. diatomicola symbionts (pink triangles, *n* = 64) and their diatom hosts (blue circles, *n* = 16). **d**, Carbon-based growth rates of symbionts (pink triangles, *n* = 64) and hosts (blue circles, *n* = 16) (black lines indicate mean; see Methods). Source Data

To confirm that *Ca*. T. diatomicola fixes N_2_, we measured the assimilation of ^15^N from ^15^N_2_ in individual *Ca*. T. diatomicola–*Haslea* symbioses using nanoscale secondary ion mass spectrometry (nanoSIMS). All investigated *Ca*. T. diatomicola cells fixed ^15^N_2_ and more than 99% of the fixed nitrogen was subsequently transferred to the diatom host, which is likely to have been facilitated by the lack of AMT transporters in *Ca*. T. diatomicola (Fig. 3a,c). As such, the symbiont fixed 100-fold more nitrogen than would be needed for its own growth, which is similar to previous reports for N_2_-fixing cyanobacteria–diatom symbioses^26^.

**Extended Data Fig. 7:**
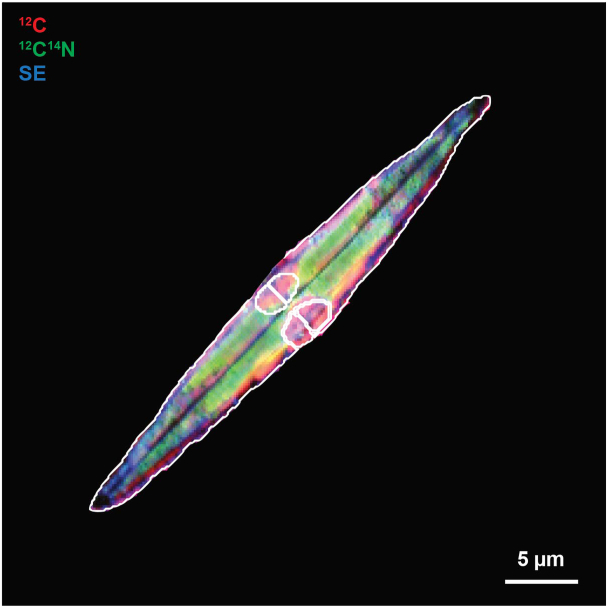
Single-cell elemental imaging of the ‘*Candidatus* Tectiglobus diatomicola’–*Haslea* symbiosis. NanoSIMS image of the distribution of carbon (^12^C, red) and nitrogen (^12^C^14^N, green) showing the carbon-rich symbionts embedded in nitrogen-rich host biomass. The image shows the same ‘*Ca*. T. diatomicola’–*Haslea* symbiosis as in Fig. 3a,b. SE, secondary electrons. Scale bar, 5 µm.

Single-cell uptake of ^13^C carbon from ^13^CO_2_, measured simultaneously with N_2_ fixation, revealed that the photosynthetic diatom in return transferred around 1% of fixed carbon to the symbiont for growth (Fig. 3b,c). The carbon supplied by the diatom might also be stored as glycogen, lipids or Calvin–Benson–Bassham-cycle products, as indicated by the carbon-rich biomass of the symbiont relative to the diatom host (Extended Data Fig. 7). Similar to nodulating rhizobia, *Ca*. T. diatomicola might store reduced carbon compounds to regulate its carbon flux and act as reductant storage^30^. Furthermore, on the basis of the similar ^13^C enrichments, both *Ca*. T. diatomicola and the *Haslea* host have comparable carbon-based growth rates (0.6 ± 0.3 and 0.8 ± 0.1 divisions per day (mean ± s.d.), respectively; Fig. 3d). When considered together with microscopic observations of dividing *Ca*. T. diatomicola–*Haslea* symbioses, this indicates the coordinated division of the symbiotic partners and vertical transmission of the symbiont (Extended Data Fig. 4a–d). Such an intricate coordination between host and symbiont growth is required for the long-term persistence and stability of a symbiosis^36^. Moreover, the fast growth of the rhizobia–diatom symbioses (mean, around 0.8 d^−1^; Extended Data Table 1) relative to the cyanobacteria–diatom symbioses (mean, around 0.2 d^−1^; Extended Data Table 1) suggests that *Ca*. T. diatomicola might contribute substantially to the nitrogen input to the oligotrophic tropical North Atlantic.

## Ecological and evolutionary implications

To assess the relative importance of the *Ca*. T. diatomicola–*Haslea* symbiosis for N_2_ fixation in surface waters of the tropical North Atlantic, we calculated their total N_2_ fixation activity on the basis of their cellular N_2_ fixation rates and abundance. Owing to the large transfer of nitrogen from the *Ca*. T. diatomicola symbiont to its host diatom (Fig. 3a,c), the biomass of both *Ca*. T. diatomicola and its *Haslea* host was considered for calculations (see Supplementary Methods). On average, N_2_ fixation rates for the *Ca*. T. diatomicola–*Haslea* symbiosis were around 650 fmol N d^−1^, which equates to around 1.5 nmol N l^−1^ d^−1^ on the basis of its in situ abundance (around 2,000 cells per litre; Extended Data Table 1). This is comparable to the combined contribution of the most abundant cyanobacteria–diatom symbioses that we observed in these waters: the cyanobacterium *Richelia*, which associates with the diatoms *Hemiaulus* and *Guinardia* (around 1.6 nmol N l^−1^ d^−1^; Extended Data Table 1). Moreover, N_2_ fixation by the *Ca*. T. diatomicola–*Haslea* symbiosis is in the same range of N_2_ fixation previously reported from this region for the most abundant cyanobacterial N_2_ fixers *Trichodesmium* and UCYN-A (up to 4 and 1.5 nmol N l^−1^ d^−1^, respectively)^10^. To our knowledge, our nanoSIMS measurements present the first direct quantitative results showing that non-cyanobacterial heterotrophic N_2_ fixers fix nitrogen in situ at rates that can account for a substantial part of the high N_2_ fixation in the tropical North Atlantic.

**Fig. 4:**
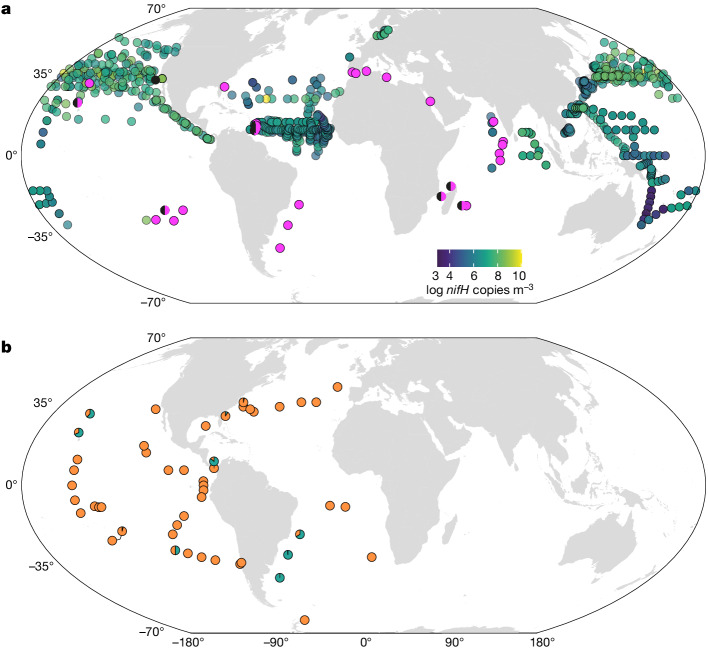
Distribution of *Candidatus* Tectiglobus diatomicola and other N_2_ fixers in the world’s oceans. **a**, Distribution of *Ca*. T. diatomicola (pink circles) and *Ca*. T. profundi (black circles) based on read detection in metagenome datasets from Tara Oceans and our own samples (see Methods and Supplementary Table 4). Black-and-pink circles are metagenomes in which both *Ca*. Tectiglobus species were detected. The abundance of *Ca*. T. diatomicola on the basis of gamma-A-specific *nifH* qPCR data is shown (circles in blue-to-yellow gradient; data from a previous study^8^). Sample locations in which gamma-A *nifH* qPCR counts were zero are shown in Extended Data Fig. 5b. **b**, Proportion of heterotrophic (orange) versus cyanobacterial (cyan) N_2_ fixers identified in a previous study^5^ (0.8–2,000 µm size fraction) in metagenome datasets from Tara Oceans.

**Extended Data Fig. 8:**
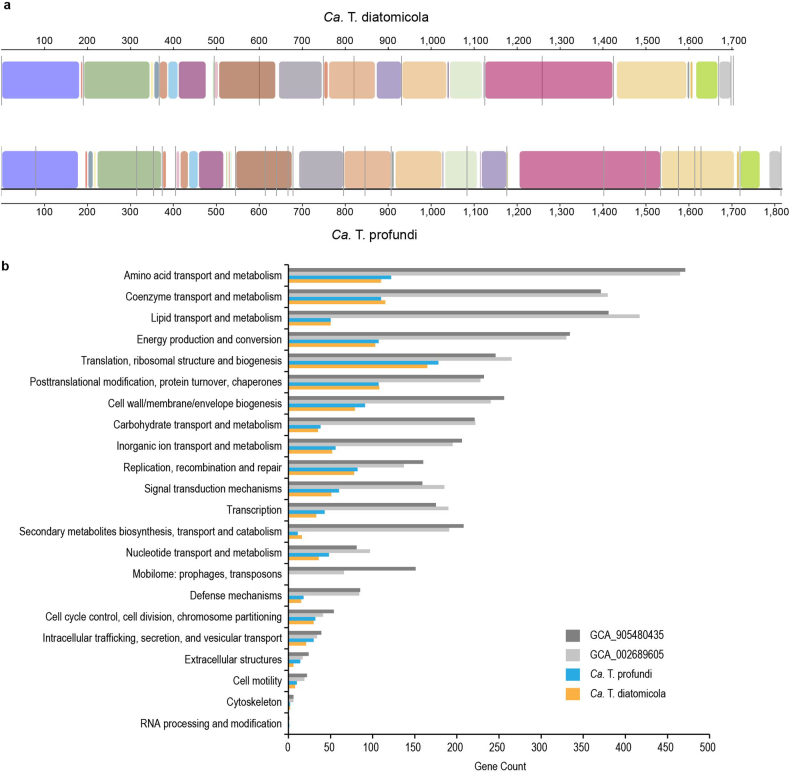
Genome comparison of ‘*Candidatus* T. diatomicola’ and ‘*Candidatus* T. profundi’. **a**, Whole-genome alignment of ‘*Ca*. T. diatomicola’ (top) and ‘*Ca*. T. profundi’ (bottom). Coloured boxes represent colinear regions present in both genomes. Grey vertical lines indicate contig boundaries. Numbers represent genomic positions in kilobase. **b**, Distribution of genes among the COG functional gene categories for the genomes of ‘*Ca*. T. diatomicola’, ‘*Ca*. T. profundi’ and their two closest relatives (GCA_905480435 and GCA_002689605). Source Data

To investigate the global distribution of this symbiosis, we retrieved sequences related to *Ca*. T. diatomicola from our own and previously published metagenomes, as well as *nifH* abundances from compilations of quantitative PCR (qPCR) data^8^. These analyses revealed that *Ca*. T. diatomicola is widespread and present in all major oligotrophic ocean regions (Fig. 4a). Notably, our metagenomic data revealed the presence of the *Ca*. T. diatomicola symbiont in regions where gamma-A was previously not reported, such as the oligotrophic South Pacific, Indian and South Atlantic Oceans. In many of these oligotrophic regions, cyanobacterial N_2_ fixers are rare^8^ and thus cannot account for the measured N_2_ fixation. We hypothesize that part of this missing nitrogen is provided by the *Ca*. T. diatomicola symbiosis. Furthermore, genomic evidence suggests that the closest relative of *Ca*. T. diatomicola, *Ca*. T. profundi, is also a widespread heterotrophic N_2_-fixing symbiont (Fig. 4a, Extended Data Figs. 3 and 8 and Supplementary Methods). A global-scale metagenomic survey^5^ indicates that heterotrophic N_2_ fixers are more common than N_2_-fixing cyanobacteria in large parts of the surface ocean (Fig. 4b). Although the contribution of these heterotrophs to oceanic N_2_ fixation remains unclear, it is noteworthy that they were frequently retrieved from the large size fraction^5^ (greater than 3 µm; Extended Data Fig. 1a), suggesting possible host association. Hence, it might be common for N_2_-fixing heterotrophs to form obligate or facultative symbioses with diatoms or other unicellular algae. By living in symbiosis with photosynthetic hosts, heterotrophic N_2_ fixers would directly fuel CO_2_ drawdown and thus contribute to oceanic carbon sequestration.

*Ca.* T. diatomicola and probably *Ca*. T. profundi represent the first host-associated members of the family *Hyphomicrobiaceae*, as well as the first marine beneficial N_2_-fixing symbionts within the order Rhizobiales. Moreover, the finding that rhizobial N_2_ fixers can form tight symbioses with unicellular algae expands the known photosynthetic hosts for Rhizobiales beyond the well-described rhizobia–legume symbiosis^17,18^. Besides *Ca*. T. diatomicola and *Ca*. T. profundi, nine other members of the *Hyphomicrobiaceae* were found to have the genomic capacity to fix N_2_, eight of which contain *nif* genes that are of alphaproteobacterial origin (Extended Data Figs. 2 and 3). The alphaproteobacterial *nifH*, *nifD*, *nifK*, *nifE*, *nifN*, *nifB* and *nifS* gene sequences from these eight *Hyphomicrobiaceae* form deeply branching sister clades to two of the major nodulating Rhizobiales *nif* clusters (the *Allorhizobium–Mesorhizobium–Rhizobium–Sinorhizobium* and the *Bradyrhizobium* clusters; Extended Data Fig. 2b). The prevalence of *nif* genes throughout the *Hyphomicrobiaceae* family indicates that their last common ancestor was capable of N_2_ fixation. This trait was subsequently lost in some members and the ancestor of *Ca*. T. diatomicola and *Ca*. T. profundi is likely to have re-acquired the capacity to fix N_2_ through horizontal gene transfer from a gammaproteobacterial donor. Such loss and subsequent re-acquisition of N_2_ fixation capacity also occurred during the evolution of the nodulating Rhizobiales^17,18^.

Because the *Hyphomicrobiaceae* evolved more than 1,000 million years ago, well before nodulating Rhizobiales lineages began to form symbioses with legume plants around 100 million years ago^17,18^, we speculate that beneficial N_2_-fixing symbioses in the Rhizobiales order evolved independently in marine environments much earlier than the nodulating species on land. Although *Ca*. T. diatomicola and the nodulating Rhizobiales evolved from one common ancestor and have similar metabolic interactions with their hosts, different degrees of host dependency have resulted in different evolutionary genome adaptations. The terrestrial nodulating rhizobial lineages form facultative symbioses with their host, and have undergone genome expansion to accommodate both a free-living and an intracellular lifestyle^18^. By contrast, the marine *Ca*. T. diatomicola has strongly reduced its genome size, in line with its proposed obligate symbiotic lifestyle. As such, the evolutionary adaptations of *Ca*. T. diatomicola are similar to those of the endosymbiotic cyanobacterium UCYN-A, which functions as an early-stage N_2_-fixing organelle^37^. It is tempting to speculate that *Ca*. T. diatomicola, which fulfils the same function in diatoms as UCYN-A does in haptophyte algae, is also in the early stages of becoming an N_2_-fixing organelle. This raises the possibility that endosymbiosis-derived N_2_-fixing organelles have originated not only from the cyanobacteria, but also from the Rhizobiales.

Nitrogen-fixing symbiotic Rhizobiales are crucial players in terrestrial productivity; they enable legumes to produce biomass through photosynthesis and consequently provide 20% of the proteins in food production (ref. ^7^ and references therein). Our results show that symbiotic marine N_2_-fixing Rhizobiales, such as *Ca*. T. diatomicola, are major contributors to oceanic N_2_ fixation and have a crucial role in sustaining marine productivity and global CO_2_ sequestration.

## Methods

### Etymology of the *Candidatus* taxa

‘*Candidatus* Tectiglobus’: Tec.ti.glo’bus. L. past part. *tectus*, hidden; L. masc. n. *globus*, a sphere; N.L. masc. n. *Tectiglobus*, a hidden sphere.

‘*Candidatus* Tectiglobus diatomicola’: di.a.to.mi’co.la. N.L. fem. n. *diatoma*, a diatom; L. suff. *-cola* (from L. masc. or fem. n. *incola*), inhabitant; N.L. masc. n. *diatomicola*, an inhabitant of diatoms.

‘*Candidatus* Tectiglobus profundi’: pro.fun’di. L. gen. n. *profundi*, from the depth of the sea, referring to the recovery of its genome from a 4,000-m-deep sediment trap.

### Sample collection and experimental set-up

Sampling was performed during two parallel cruises on board RV *Maria S. Merian* (cruise MSM89; Bridgetown, Barbados–Bridgetown, Barbados) and RV *Meteor* (cruise M161; Bridgetown, Barbados–Ponta Delgada, Azores, Portugal) in January–February 2020 in the western tropical North Atlantic. Samples were obtained from Niskin rosette samplers equipped with conductivity, temperature and depth (CTD) systems. At each station, CTD casts were performed to obtain surface water (around 10 m) for dawn-to-dawn incubation experiments of CO_2_ and N_2_ fixation rates using stable isotope tracers. At the beginning of the incubation experiments, subsamples for DNA and RNA sequencing and FISH were taken. Samples for DNA and RNA sequencing were taken by sequential filtration of 10 l of seawater through 10-µm and 3-µm polycarbonate filters (Isopore, 47 mm diameter) followed by two parallel 0.22-µm Sterivex filters (5 l was filtered through each of the two 0.22-µm filters; all filters from Merck). After filtration, filters were flash-frozen in liquid nitrogen and stored at −80 °C until processing. At the end of the approximately 24-h incubation experiments, subsamples for measurements of bulk rate and for FISH and single-cell analyses were taken. On the RV *Meteor* cruise, additional DNA and RNA samples were collected at the end of the approximately 24-h incubation period through sequential size filtration of around 3 l of incubated seawater. Samples for FISH and nanoSIMS were preserved with methanol-free paraformaldehyde solution (1% w/v final concentration) either for around 24 h at 4 °C or for a few hours at 4 °C followed by 0.5 h at room temperature. Preserved samples were subsequently filtered onto polycarbonate filters (Isopore, 0.2 µm pore size, 25 mm diameter); all samples intended for nanoSIMS analyses were filtered onto gold (Au)-coated polycarbonate filters. Filters were subsequently rinsed with ultrapure water (MilliQ), dried and stored at −20 °C until further analyses.

### Metagenomic and metatranscriptomic sequencing

Samples from a total of eight stations were selected for DNA and RNA extractions and subsequent long- and short-read metagenomic and metatranscriptomic sequencing. All library preparation steps and sequencing were performed at the Max Planck Genome Centre (http://mpgc.mpipz.mpg.de/home/). See Supplementary Methods for details of samples, DNA and RNA extraction protocols, library preparation for short- and long-read sequencing and quality trimming.

### Recovery and annotation of the *Candidatus* Tectiglobus diatomicola genome

The genome of *Ca*. T. diatomicola was reconstructed from a deeply sequenced short-read metagenomic sample (around 315 Gb, 3–10 µm size fraction from surface water after 24 h of incubation, station 4 from the M161 cruise) together with long-read metagenomes from six stations from the MSM89 cruise (all size fractions from surface water before incubations), as follows. Raw metagenomic short reads were trimmed using Trimmomatic^38^ v.0.39 (ILLUMINACLIP:TruSeq3-PE.fa:2:30:10, LEADING:3, TRAILING:3, SLIDINGWINDOW:4:15, MINLEN:36) and assembled using MEGAHIT^39^ v.1.2.9. To reduce the size of the assembly (33 million contigs, totalling around 17.7 Gb), it was filtered to retain only contigs with a length of more than 2 kb, 25–40% GC content and a coverage of 29–44; the latter two parameters were chosen on the basis of a preliminary reconstruction of the *Ca*. T. diatomicola genome (for further details, see Supplementary Methods). The remaining 8,218 contigs were visualized in anvi’o^40^ v.7.1, and those with high similarity to the previously reconstructed *Ca*. T. diatomicola genome were identified using blastn (BLAST+ (ref. ^41^) v.2.9.0). Using this approach, a tightly clustered group of 189 contigs, most of which matched with contigs of the previously reconstructed *Ca*. T. diatomicola, was identified in anvi’o^40^ v.7.1. The 189 contigs were then iteratively extended and refined, and one iteration included the following steps (see ‘Code availability’). Long and short metagenomic reads were mapped onto contigs using minimap2 (ref. ^42^) v.2.22-r1101 (‘-ax map-hifi’ for long reads and ‘-ax sr --score-N 2’ for short reads) and the sam mapping files were converted into bam format using SAMtools^43^ v.1.14 (‘samtools view’) and filtered to retain only reads that mapped with more than 98% identity (and more than 80% of the read length for short reads only) using CoverM v.0.6.1 (https://github.com/wwood/CoverM). The remaining mapped long and short reads were converted into fasta format using SAMtools^43^ v.1.14 (‘samtools fasta’). Short-read pairs in which only a single read was mapped were completed using seqkit^44^ v.2.3.0. Subsequently, mapped long and short reads were assembled together using SPAdes^45^ v.3.15.3 (‘--isolate -k 21,33,55,77,99,111’, mapped long reads were supplied using ‘-s’ and scaffolds of the previous iteration were supplied using ‘--trusted-contigs’) and the assembled scaffolds were filtered to retain only scaffolds of at least 1 kb. The filtered scaffolds were then used as input for the next iteration. After 23 iterations, the scaffolds were manually inspected and refined using anvi’o^40^ v.7.1, resulting in the current *Ca*. T. diatomicola genome. Completeness and redundancy were estimated using CheckM2 (ref. ^46^) v.0.1.2 and taxonomy was assigned using GTDB-TK^47^ v.2.1.0 and the Genome Taxonomy Database (GTDB)^16^ v.r214.

The *Ca*. T. diatomicola genome was at first annotated using Prokka^48^ v.1.14.6. Additional information about gene functions was sourced from the RAST web server^49^ (https://rast.nmpdr.org/) and through DIAMOND^50^ v.2.0.8 similarity searches against the KEGG^51^ v.58 and eggNOG^52^ v.4.5 databases using the utility script ‘sqm_annot.pl’ from the SqueezeMeta metagenomics pipeline^53^ v.1.6.2. The utility script ‘sqm_annot.pl’ was further used for taxonomic annotation of all coding sequences using a last common ancestor approach^53^. For genes of interest on the *nif* cluster encoding contig, the taxonomic origin was further investigated using phylogenetic analyses and/or by manual inspection of their best blast hits (see Supplementary Methods). Highly transcribed genes (top 20%) were further inspected using the InterPro web server^54^ v.95.0-97.0 (https://www.ebi.ac.uk/interpro/result/InterProScan) and searches against the NCBI-nr database^55^ using the NCBI BLAST web server (https://blast.ncbi.nlm.nih.gov/Blast.cgi).

### Genome comparisons

The gene content of the two *Ca*. Tectiglobus genomes was compared to that of two closely related MAGs (GCA_905480435 and GCA_002689605). The genes for each genome were annotated with COG identifiers using anvi’o^40^ v.7.1 (https://merenlab.org/2016/10/25/cog-annotation/) and genes within each COG category were summed. Whole-genome alignment and identification of blocks of conserved regions within the two *Ca*. Tectiglobus genomes were performed using mauve^56^ (development snapshot 2015-02-26).

### Phylogenetic analyses

A maximum likelihood phylogenetic tree of *Ca*. T. diatomicola, *Ca*. T. profundi and all Rhizobiales and Parvibaculales (outgroup) genomes from the GTDB^16^ (v.r214), as well as HBD_Alpha_05 (a marine MAG from the *Hyphomicrobiaceae* family also containing *nif* genes^5^) was calculated on the basis of 16 ribosomal proteins^57^, using muscle^58^ v.3.8.1551 for alignment and FastTree^59^ v.2.1.11 for tree calculation. The resulting tree was visualized in iToL^60^ v.6.8.1. For further details and the phylogeny of marker proteins (NifH, NifD, NifK, NifE, NifN, NifB, NifS and CcoN), see Supplementary Methods.

### *Candidatus* Tectiglobus diatomicola transcriptome analysis

To obtain gene transcription information for *Ca*. T. diatomicola, all sequenced metatranscriptome reads were combined and mapped to the *Ca*. T. diatomicola genome using BWA-MEM^61^ v.0.7.17-r1188, and the resulting mapping files were filtered requiring at least 95% sequence identity and at least 80% of the read to align (mapping and filtering were done through CoverM v.0.6.1). Gene counts were generated using featureCounts^62^ v.2.0.1 and TPM values for protein-coding genes were calculated as previously described^63^. The genome plot (Fig. 2a) including TPM values was generated using BRIG^64^ v.0.95 and DNAPlotter^65^ v.18.1.0.

### Global abundance of *Candidatus* Tectiglobus diatomicola and *Candidatus* Tectiglobus profundi

To determine the global distribution and abundance of *Ca*. T. diatomicola and *Ca*. T. profundi, we analysed their presence in metagenomes and in publicly available qPCR data. To this end, we used metagenomes from the Tara Oceans campaign (total of 1,241 metagenomes from projects PRJEB4352, PRJEB1787, PRJEB9691 and PRJEB9740) and metagenomes that we obtained from the tropical North Atlantic and the South Pacific gyre (see Supplementary Table 4). The contigs for each of the two genomes were concatenated (not including the regions encoding the rRNA gene clusters to reduce non-specific read recruitment) and metagenomic reads were mapped using bbmap v.38.70 (https://sourceforge.net/projects/bbmap/) with a minimum identity threshold of 90%. We only considered each genome to be present in a metagenome when the breadth of coverage (fraction of the genome covered by at least one read) was close to the expected breadth following a previously reported formula^66^:

Expected breadth = 1 – e^(−0.883 × coverage)^ (Extended Data Fig. 5c,d).

In addition, we downloaded gamma-A (that is, *Ca*. T. diatomicola) *nifH* qPCR data^67^ from a previous study^8^ (referred to as NCD_gammaA_nifH_gene and NCD_g24774A11_nifH_gene, around 2,500 data points), added a pseudocount of 1 to the *nifH* copy numbers, log-transformed the counts and filtered out all samples with a count value of 0. We then plotted the coordinates of all metagenomes in which either of the two *Ca*. Tectiglobus genomes were detected together with the log-transformed *Ca*. T. diatomicola *nifH* qPCR counts on a world map using R^68^ (Fig. 4). qPCR samples in which *Ca*. T. diatomicola (gamma-A) *nifH* had a count value of 0 were plotted separately (Extended Data Fig. 5b).

### Software for bioinformatics analyses

Further software that was used during the analysis of the sequencing data that is described in the Supplementary Information: hifiasm-meta^69^ v.0.2-r043 for the assembly of long-read metagenomes; CompareM v.0.1.2 (https://github.com/dparks1134/CompareM) for the calculation of average amino acid identity between *Ca*. T. diatomicola and closely related genomes; fastANI^70^ v.1.33 for the calculation of average nucleotide identity between preliminary MAGs; USEARCH^71^ v.11.0.667 for clustering sequences on the basis of similarity before phylogenetic tree constructions; MAFFT^72^ v.7.505 for calculating and trimAl^73^ v.1.4.1 for trimming multiple sequence alignments; ModelFinder^74^ for predicting best-fitting models; and UFBoot2 (ref. ^75^) for calculating ultrafast bootstraps during the construction of maximum likelihood trees with IQ-TREE^76^ v.2.2.0.3 and v.2.2.2.7. The following software was used as part of the SqueezeMeta metagenomics pipeline^53^ v.1.6.2: Barrnap 0.9-dev (https://github.com/tseemann/barrnap) for the prediction of ribosomal RNAs; the RDP classifier^77^ v.2.10.2 for the taxonomic classification of predicted 16S rRNA sequences; prodigal^78^ v.2.6.3 for gene prediction; HMMER v.3.1b2 (http://hmmer.org/) for HMM homology searches against the Pfam database^79^; Bowtie2 (ref. ^80^) v.2.3.4.1 for mapping short reads; MetaBAT 2 (ref. ^81^) v.2.12.1, MaxBin 2.0 (ref. ^82^) and CONCOCT^83^ v1.1.0 for the binning of contigs into MAGs; DAS Tool^84^ v.1.1.1 for integrating the results from the three binning tools; and bbduk v38.87 (https://sourceforge.net/projects/bbmap/) for trimming of metatranscriptomic reads.

### Bulk rates of CO_2_ and N_2_ fixation

Rates of CO_2_ and N_2_ fixation were determined as previously described^10,85^ (with a detailed description in the Supplementary Methods). Stable isotope incubations (^15^N-N_2_ and ^13^C-DIC (dissolved inorganic carbon)) were performed in triplicate for 24 h (dawn to dawn). Bottles were incubated in on-deck incubators continuously flushed with surface seawater, with simulated light conditions^86^. After around 24 h, subsamples were taken for elemental and isotopic biomass analyses as well as FISH and nanoSIMS analyses. Fixation rates were calculated on the basis of the incorporation of ^13^C and ^15^N into biomass (that is, the change in isotopic composition) for both bulk and single-cell activities (Supplementary Methods).

### Visualization and abundance of *Candidatus* Tectiglobus diatomicola

To visualize the newly identified *Ca*. T. diatomicola, we designed FISH probes targeting the 16S rRNA^87,88^ (Supplementary Methods). In total, four FISH probes were designed: two specifically targeting *Ca*. T. diatomicola (Hypho825 and Hypho638) and two with a broader coverage, targeting many members of the *Hyphomicrobiaceae* (Hypho1147) and several members of the *Hyphomicrobiaceae* genera *Hyphomicrobium*, *Filomicrobium* and *Pedomicrobium* (Hypho734) (see Extended Data Table 2 and Supplementary Methods). The optimal formamide concentrations for these new probes were tested using Clone-FISH^89^ (Supplementary Methods).

The *Ca*. T. diatomicola cells were visualized using catalysed reporter deposition-FISH (CARD-FISH) with the four new horseradish peroxidase (HRP)-labelled probes either alone or in combination (double hybridization) and together with helpers or competitors to increase signal intensity (Extended Data Table 2). CARD-FISH was performed as previously described^90^. Microscopy was performed using a Zeiss Axio Imager.M2 wide-field epifluorescence microscope equipped with a Zeiss Axiocam 506 mono camera and Zeiss ZEN 3.2 blue edition software, a Zeiss LSM 780 confocal laser scanning microscope equipped with Zeiss Elyra PS.1 super-resolution-structured illumination microscopy and a laser microdissection (LMD) microscope (LMD 7000, Leica). For better identification of the diatom host of *Ca*. T. diatomicola, host diatoms containing FISH-positive cells were visualized by SEM with a FEI Quanta 250 FEG ESEM (Thermo Fisher Scientific, FEI) (see Supplementary Methods). Abundances of the *Ca*. T. diatomicola–*Haslea* symbiosis were determined as the number of diatom hosts containing FISH-positive cells from filter pieces representing around 25 ml of sampled water (Extended Data Table 1).

### Abundance of free-living and host-associated cyanobacterial N_2_ fixers

Free-living (*Trichodesmium*, *Crocosphaera*) and host-associated (diatom-associated *Richelia*) cyanobacterial N_2_ fixers were identified by morphology and chlorophyll *a*- and/or phycoerythrin autofluorescence using an LMD microscope (LMD 7000, Leica). *Richelia* were found associated with the diatoms *Guinardia*, sometimes also referred to as *Rhizosolenia*, and *Hemiaulus*. Abundances were determined as the number of *Richelia*-containing host diatoms. Abundances of *Trichodesmium* were determined by measuring the total (summed) length of all free trichomes, and by dividing the total trichome length by the average cell length. Abundances of *Crocosphaera* cells were determined by direct cell counts. Abundances were determined from whole filters representing around 300–360 ml of sampled water (Extended Data Table 1).

### Single-cell N_2_-fixation activities using nanoSIMS

Au-sputtered filters from the end of the stable isotope incubations were used for nanoSIMS (nanoSIMS 50L, CAMECA) to measure the single-cell carbon and nitrogen isotopic composition and to determine the single-cell CO_2_ and N_2_ fixation activities of *Ca*. T. diatomicola and their *Haslea* hosts as well as *Richelia* and their diatom hosts (*Hemiaulus* and *Guinardia*) (Extended Data Table 1). Both target symbioses were visualized as described above and subsequently marked using an LMD microscope (LMD 7000, Leica). NanoSIMS analyses were performed as previously described^90^ and details are provided in the Supplementary Methods. The isotopic ratios (^13^C/^12^C and ^12^C^15^N/^12^C^14^N) of regions of interest were determined by overlaying epifluorescence images of FISH-positive cells and their host diatom (for *Ca*. T. diatomicola–*Haslea* symbiosis) or brightfield and autofluorescence images (diatom-associated *Richelia*) with the nanoSIMS images (secondary electrons). For the elemental imaging (Extended Data Fig. 7), carbon (^12^C), nitrogen (^12^C^14^N) and secondary electrons were overlaid; for this image only, background signals were removed. Cellular rates and abundances were combined to determine the absolute contributions of the *Ca*. T. diatomicola–*Haslea* symbiosis and diatom-associated *Richelia* to the bulk N_2_ fixation rate as previously described^10^. For both the *Ca*. T. diatomicola–*Haslea* symbiosis and the diatom-associated *Richelia*, the amount of nitrogen recovered in the diatom was also taken into account. Carbon-based growth rates for individual cells as well as whole symbioses were determined using the equation provided in a previous report^10^ and mass-balancing host and symbiont. Single-cell activities and contributions in our study can be considered conservative because ^13^C/^12^C and ^15^N/^14^N ratios can be diluted during sample preparation, which can lead to underestimation^91–93^.

### Statistics and reproducibility

For Fig. 1b,c, the correlative SEM and fluorescence (confocal as well as epifluorescence) images are representative of a total of 11 diatoms from surface waters of 3 independent environmental samples. Additional fluorescence images (Fig. 1c) were obtained from a total of 27 diatoms from 6 independent environmental samples.

For Fig. 3a,b, the correlative nanoSIMS images are representative of a total of 16 diatoms (containing a total of 64 symbionts) from 3 independent environmental samples.

For Extended Data Fig. 4, the correlative fluorescence (confocal as well as epifluorescence) and SEM images (Extended Data Fig. 4a–d) are representative of a total of 13 diatoms, which contained more than 4 symbionts, from 5 independent environmental samples. The NON338-probe image (Extended Data Fig. 4e) is representative of a total of 32 diatoms from 3 independent environmental samples; however, chloroplasts were not always visible. Extended Data Fig. 4f, showing *Ca*. T. diatomicola cells after hybridization with a specific oligonucleotide probe in close vicinity to the H-shaped nucleus and bilobed chloroplasts, is representative of a total of 18 diatoms from 3 independent environmental samples. The SEM image showing a whole diatom (Extended Data Fig. 4g) is representative of a total of 19 diatoms from 2 independent environmental samples. Extended Data Fig. 4h–k show a selection of magnified images that helped with the identification of the diatom host.

For Extended Data Fig. 7, one diatom was analysed.

### Reporting summary

Further information on research design is available in the Nature Portfolio Reporting Summary linked to this article.

## Online content

Any methods, additional references, Nature Portfolio reporting summaries, source data, extended data, supplementary information, acknowledgements, peer review information; details of author contributions and competing interests; and statements of data and code availability are available at 10.1038/s41586-024-07495-w.

### Supplementary information

Supplementary Information This file contains Supplementary Methods, Supplementary Discussion and Supplementary References.

Reporting Summary

Supplementary Table 1 This file contains Supplementary Table 1, which lists the genome annotation and transcriptome counts of ‘*Ca.* T. diatomicola’.

Supplementary Table 2 This file contains Supplementary Table 2, which lists *Haslea* spp. contigs identified in the metagenome assembly.

Supplementary Table 3 This file contains Supplementary Table 3, which lists the presence/absence of KEGG modules in the two ‘*Ca.* Tectiglobus’ (diatomicola and profundi) genomes and genomes of other *Hyphomicrobiaceae*.

Supplementary Table 4 This file contains Supplementary Table 4, which lists metagenomes used for the global distribution analysis.

Supplementary Table 5 This file contains Supplementary Table 5, which lists the genome annotation of ‘*Ca.* T. profundi’.

Supplementary Data 1–9 These are tree files corresponding to the phylogenetic trees shown in Fig.1 and Extended Data Figs. 2 and 3.

### Source data

Source Data Fig. 3

Source Data Extended Data Fig. 1

Source Data Extended Data Fig. 3

Source Data Extended Data Fig. 5

Source Data Extended Data Fig. 8

## Figures and Tables

**Extended Data Table 1:**
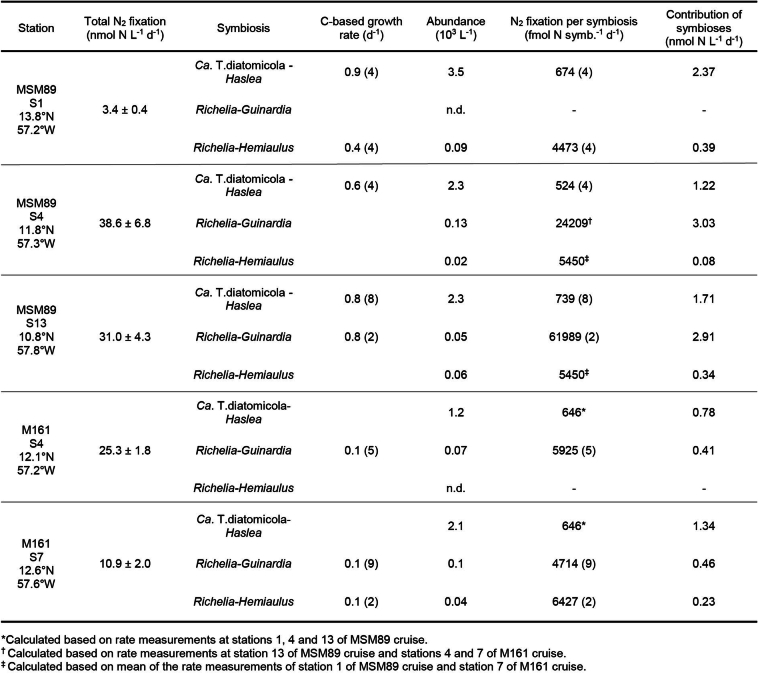
Abundances and single-cell activities of diatom symbioses in the tropical North Atlantic

N_2_ fixation rates and abundances of ‘*Ca*. T. diatomicola’–*Haslea*, *Richelia–Guinardia* and *Richelia–Hemiaulus* symbioses and their contribution to the total N_2_ fixation rates at five different stations in the tropical North Atlantic. n.d. not detected. The number of diatom symbioses measured on nanoSIMS is shown in parentheses.

**Extended Data Table 2:**
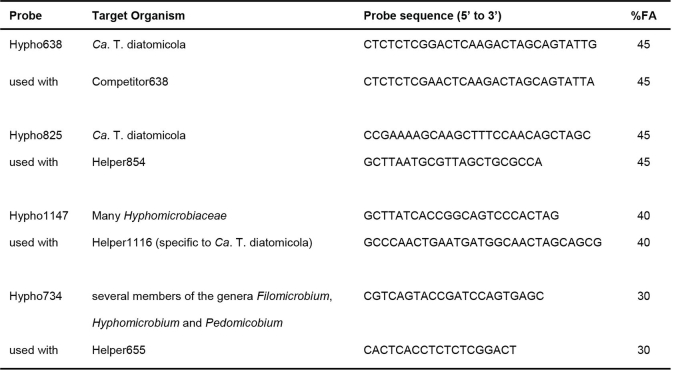
Summary of 16S-rRNA-targeted oligonucleotide probes used for FISH

Probe names are listed with their target organisms, sequences and percentage of formamide (%FA) used during hybridization.

## Data Availability

Read data from metagenomic analyses pertaining to *Ca*. T. diatomicola have been deposited at the NCBI under BioProject accession number PRJNA1036431, including the MAGs of *Ca*. T. diatomicola and *Ca*. T. profundi under the accession numbers JAZDSJ000000000 and DAWWJP000000000, respectively. RNA-sequencing data can be found under the same BioProject number with the accession numbers SRR26695118, SRR26695119 and SRR26695121–SRR26695130. The publicly available sequences used for phylogenetic tree construction and genome comparison can be found at the GTDB (https://gtdb.ecogenomic.org/) under the accession numbers given in Supplementary Data 1–9 (tree file for each tree). Publicly available MAGs from a previous study^5^ can be found at https://figshare.com/articles/dataset/Marine_diazotrophs/14248283. Tara Oceans metagenomes used in this study can be found at https://www.ncbi.nlm.nih.gov/bioproject/173486 with the following BioProject accessions used in this study: PRJEB4352 (size fractions for protists), PRJEB1787 (size fractions for prokaryotes), PRJEB9691 (size fractions for protists from polar circle samples) and PRJEB9740 (size fractions for prokaryotes from polar circle samples). For the reconstruction of the MAG of *Ca*. T. profundi, the metagenomic data that we used can be found at the GTDB (https://gtdb.ecogenomic.org/genome?gid=GCA_013214245.1; original MAG) and at the Sequence Read Archive under https://www.ncbi.nlm.nih.gov/bioproject/PRJNA482655 with the accession numbers SRR7648332, SRR7648341, SRR7648350, SRR7632647 and SRR7648334 (metagenomes used for MAG reconstruction). Publicly available qPCR data from a previous study^8^ can be found at 10.6084/m9.figshare.21677687.v3. Additional databases used in this study can found at the following links: eggNOG: http://eggnog45.embl.de/download/eggnog_4.5/data/NOG/; ncbi-nr database: https://ftp.ncbi.nlm.nih.gov/blast/db/FASTA/nr.gz; pfam database: https://www.ebi.ac.uk/interpro/download/pfam/; and kegg database: http://andes.cnb.csic.es/SqueezeMeta/kegg.db.gz. Source data are provided with this paper.

## Code availability

Custom scripts for iterative bin refinements are deposited at https://github.com/bresyd/mag_refinement/tree/main.
