# Supplementary material for: Rhizobia–diatom symbiosis fixes missing nitrogen in the ocean
Source: Nature. 2024 May 9;630(8018):899–904. doi: 10.1038/s41586-024-07495-w (PMC11208148; doi:10.1038/s41586-024-07495-w)
Supplement: Supplementary file 1 — This file contains Supplementary Methods, Supplementary Discussion and Supplementary References. [file 41586_2024_7495_MOESM1_ESM.pdf]

nature portfolio

https://doi.org/10.1038/s41586-024-07495-w

Supplementary information

Rhizobia–diatom symbiosis fixes missing nitrogen in the ocean

In the format provided by the
authors and unedited

Supplementary Information

Rhizobia–diatom symbiosis fixes missing nitrogen in the ocean

Authors: Bernhard Tschitschko[1,6], Mertcan Esti[1], Miriam Philippi[1,7], Abiel T. Kidane[1], Sten Littmann[1], Katharina Kitzinger[1,2], Daan R. Speth[1,8], Shengjie Li[1], Alexandra Kraberg[3], Daniela Tienken[1], Hannah K. Marchant[1,4], Boran Kartal[1,5], Jana Milucka[1], Wiebke Mohr[1], Marcel M. M. Kuypers[1,*]

Affiliations

[1] Max Planck Institute for Marine Microbiology, Celsiusstraße 1, 28359 Bremen, Germany
[2] Centre for Microbiology and Environmental Systems Science, Division of Microbial Ecology, University of Vienna, Djerassiplatz 1, 1090 Vienna, Austria
[3] Alfred Wegener Institute - Helmholtz-Centre for Polar and Marine Research, Am Handelshafen 12, 27570 Bremerhaven, Germany
[4] MARUM – Centre for Marine Environmental Sciences, University Bremen, Leobener Str. 8, 28359 Bremen, Germany
[5] School of Science, Constructor University, Bremen, Germany
[6] Present address: Department of Microbiology, University of Innsbruck, Technikerstraße 25d, 6020 Innsbruck, Austria
[7] Present address: Alfred Wegener Institute - Helmholtz-Centre for Polar and Marine Research, Am Handelshafen 12, 27570 Bremerhaven, Germany
[8] Present address: Centre for Microbiology and Environmental Systems Science, Division of Microbial Ecology, University of Vienna, Djerassiplatz 1, 1090 Vienna, Austria

[*] For correspondence: mkuypers@mpi-bremen.de

Contents:

Supplementary Methods
Supplementary Discussion
Supplementary References

Supplementary Methods

Metagenomic and -transcriptomic sequencing

Samples from eight stations were selected for DNA/RNA extraction and subsequent metagenomic and metatranscriptomic sequencing. These included three size fractions (0.22-3 µm, 3-10 µm and > 10 µm) from surface water from six stations from the MSM89 cruise (MSM89-S1, 2, 4, 10, 13 and 14) and two stations from the M161 cruise (M161-S4 and 7). DNA and RNA was co-extracted using the ZR-Duet DNA/RNA MiniPrep Kit (Zymo Research) and all library preparation steps and sequencing were performed at the Max Planck Genome Centre Cologne. Short-read metagenomic sequencing was performed on all samples, except 0.22-3 µm size fractions of the MSM89 cruise, using the Illumina HiSeq3000 platform with 2 x 150 bp paired-end reads with a sequencing depth of ~15-20 Gb per sample. Long-read metagenomic sequencing was performed on all samples from the MSM89 cruise using the Ultra-Low (>10 µm and 3-10 µm size fractions due to low amount of input DNA) and Low (0.22-3 µm size fractions) DNA Input Workflow for SMRT Sequencing (Pacific Biosciences) and sequenced using a PacBio Sequel II, multiplexing two samples per SMRT cell. Metatranscriptomic sequencing was done on the > 10 µm and 3-10 µm size fractions from all stations of the MSM89 cruise. Total RNA libraries were sequenced with the Illumina HiSeq3000 platform with 1 x 150 bp single reads with a sequencing depth of ~30 Gb per sample. Raw metagenomic and metatranscriptomic short reads were trimmed using Trimmomatic[38] v0.39 (ILLUMINACLIP:TruSeq3-PE.fa:2:30:10, LEADING:3, TRAILING:3, SLIDINGWINDOW:4:15, MINLEN:36) and bbduk v38.87 (ktrim=l, trimq=10, qtrim=rl, minlength=50, mink=11) (BBMap – Bushnell B. – sourceforge.net/projects/bbmap/), respectively.

Initial *Ca.* T. diatomicola genome reconstruction

Ca. T. diatomicola *nifH* genes were first identified in a read-level analysis of the PacBio long-reads using the "sqm_longreads.pl" script, which is part of the SqueezeMeta metagenomics pipeline[53] v1.6.2. This script performed DIAMOND[50] v2.0.8 blastx searches against the NCBI-nr[55] (release 247), KEGG[51] v58 and eggNOG[52] v4.5 databases. The predicted protein sequences were then searched for the presence of the full-length Gamma-A *nifH*[97] using blastp[41], resulting in a number of matching proteins (> 99% sequence identity over the full length). One sample that contained multiple Gamma-A *nifH* encoding reads was then chosen for an in-depth analysis (MSM89-S1, 3-10 µm size fraction). The PacBio long-reads of this sample were assembled into contigs using the long-read metagenome assembler hifiasm-meta[69] v0.2-r043. To reduce the loss of sequence information, unassembled reads were added to the contigs. In detail, long reads were mapped to contigs using minimap2 (ref. [42]) v2.22-r1101 and mapped reads were removed from the mapping file using SAMtools[43] ('samtools view –f'). The fasta sequences of the remaining unmapped reads were subsequently extracted using the script reformat.sh v38.70 (BBMap – Bushnell B. – sourceforge.net/projects/bbmap/) and added to the assembled contigs. The combined contigs and reads were then analyzed using the SqueezeMeta metagenomics pipeline[53] v1.6.2 including taxonomic and functional annotation of all reads and contigs as well as binning into metagenome-assembled genomes (MAGs).

The SqueezeMeta metagenomics pipeline performed the following analyses: ribosomal RNA prediction using Barrnap 0.9-dev (https://github.com/tseemann/barrnap); taxonomic classification of predicted 16S rRNA sequences using the RDP classifier[77] v2.10.2; gene prediction using prodigal[78] v2.6.3; functional and taxonomic annotation of genes using DIAMOND[50] v2.0.8 similarity searches against NCBI-nr[55] release 247, KEGG[51] v58 and eggNOG[52] v4.5 databases; as well as HMM homology searches against the Pfam database[79] using HMMER v3.1b2 (http://hmmer.org/). Regions with no predicted genes were further annotated using DIAMOND[50] v2.0.8 blastx searches against NCBI-nr[55]. Final functional and

taxonomic annotation of genes was performed using the SqueezeMeta last common ancestor (LCA) and fun3 algorithms. Taxonomic annotation of contigs was then performed on a consensus approach based on the annotation of all encoded genes[53]. Mapping of Illumina short reads to the combined long reads and contigs was done using Bowtie2 (ref. [80]) v2.3.4.1 and subsequent binning of MAGs was done using MetaBAT 2 (ref. [81]) v2.12.1, MaxBin 2.0 (ref. [82]) and CONCOCT[83] v1.1.0. Results from the three binning tools were then integrated using DAS Tool[84] v1.1.1 and MAG statistics were computed using CheckM[98]. In the resulting MAG collection, one low-quality MAG (total size of ~220 Kb, 7.6% completeness, 0% redundancy) assigned to the *Hyphomicrobiaceae* contained the Gamma-A *nifH* gene. The relative abundance of this MAG across all short-read metagenomes was calculated using CoverM v0.6.1 (https://github.com/wwood/CoverM) and its abundance was highest in the larger size fractions (3-10 µm and > 10 µm) of samples from Stations 4 and 7 of the M161 cruise.

Metagenomic short reads from the two stations in which the *Hyphomicrobiaceae* MAG had highest abundance (MSM161-S4 and S7) were then co-assembled using MEGAHIT[39] v1.2.9. The assembled contigs were analyzed using the SqueezeMeta metagenomics pipeline[53] v1.6.2 as described above. The resulting MAGs were then searched for the presence of a MAG that was similar to the previously identified low-quality *Hyphomicrobiaceae* MAG using fastANI[70] v1.33, revealing a better-quality MAG (total size of ~1.1 Mb, ~50% completeness, 0.5% redundancy), which was also assigned to the *Hyphomicrobiaceae*. This MAG was then iteratively extended and refined using a custom script described in the main Methods section (see code availability statement), resulting in an improved MAG of ~1.25 Mb, 76% completeness and 0% redundancy based on CheckM2 (ref. [46]) v0.1.2 estimates, which is particularly accurate for organisms with reduced genomes. The contigs of this improved MAG were used as queries during the reconstruction of the final *Hyphomicrobiaceae* MAG, ultimately called *Ca.* T. diatomicola.

Recovery of *Haslea* spp. in metagenomic contigs

To investigate the presence of *Haslea* spp. diatoms in our metagenomes, the large assembly (contigs > 1 Kb) generated for the recovery of the final *Ca*. T. diatomicola genome was annotated using the SqueezeMeta metagenomics pipeline[53] v1.6.2 as described above. Using the contig consensus annotation, 17 contigs assigned to the genus *Haslea* were identified (Supplementary Table 2). At the time of our analysis, the NCBI protein database contained < 3,000 sequences from only a few *Haslea* species, and almost all of them represented chloroplast or mitochondrial sequences. It is thus likely that our assembly contained more *Haslea* contigs, which escaped detection.

Genome refinement of *Ca.* T. profundi

The initial MAG (GCA-013214245) of the bacterium that we describe as *Ca.* T. profundi was originally retrieved from a 4000-m deep sediment trap in the North Pacific[99]. Since this initial MAG did not contain genes for nitrogenase, we iteratively extended and refined it with the aim of recruiting nitrogenase genes potentially missed in the original study[99]. Prior to the genome refinement, the raw metagenomic short reads from sixty-three metagenomes[99,100] were downloaded and trimmed using Trimmomatic[38] v0.39 (ILLUMINACLIP:TruSeq3-PE.fa:2:30:10, LEADING:3, TRAILING:3, SLIDINGWINDOW:4:15, MINLEN:36). The trimmed reads were mapped to GCA-013214245 using BBMap v38.98 (BBMap – Bushnell B. – sourceforge.net/projects/bbmap/) with a 95% identity cutoff and the abundance of GCA-013214245 across metagenomes was obtained using CoverM v0.6.1 (https://github.com/wwood/CoverM). The five samples in which GCA-013214245 had highest abundance were then used to iteratively extend and refine the genome using a custom script described in the main Methods section (see code availability statement). After 17 iterations, the scaffolds were manually inspected and refined using anvi'o[40] v7.1, resulting in the refined *Ca.* T. profundi genome. Completeness (100%) and redundancy (0%) of the *Ca.* T. profundi genome

was estimated using CheckM2 (ref. [46]) v0.1.3. The *Ca*. T. profundi genome was annotated using Prokka[48] v1.14.6. Additional information about gene functions was sourced through DIAMOND[50] v2.0.8 similarity searches against the KEGG[51] v58 and eggNOG[52] v4.5 databases using the utility script 'sqm_annot.pl from the SqueezeMeta metagenomics pipeline[53] v1.6.2.

Abundance of cyanobacterial and heterotrophic N_2 fixers in the tropical North Atlantic

The relative abundance of eight cyanobacterial and forty heterotrophic N_2 fixers[5,101] as well as the two *Ca.* Tectiglobus genomes was assessed in the metagenomes we generated from the tropical North Atlantic. Relative abundance of genomes in the metagenomes was calculated based on mapped reads using CoverM v0.6.1 (https://github.com/wwood/CoverM), requiring ≥ 95% sequence identity and ≥ 80% of the read to align.

Phylogenetic analyses

A maximum likelihood phylogenetic tree of *Ca*. T. diatomicola and *Ca*. T. profundi within Rhizobiales was calculated based on sixteen ribosomal proteins[57] as follows: 2,376 Rhizobiales and 55 Parvibaculales (outgroup) genomes were retrieved from the genome taxonomy database (GTDB)[16] version r214. To this collection, the genomes of *Ca*. T. diatomicola and *Ca*. T. profundi were added. For each genome, an anvi'o[40] database (anvi'o development version) was then generated, including gene calling using prodigal[78] v2.6.3 and identification of the 71 bacterial marker genes included in anvi'o using HMMER v3.3.2 (http://hmmer.org/). The sixteen ribosomal proteins (Ribosomal_L2, Ribosomal_L3, Ribosomal_L4, Ribosomal_L5, Ribosomal_L6, Ribosomal_L14, Ribosomal_L16, Ribosomal_L18p, Ribosomal_L22, Ribosomal_L24, Ribosomal_S3_C, Ribosomal_S8, Ribosomal_S10, Ribosomal_S17, Ribosomal_S19) were extracted, aligned with muscle[58] v3.8.1551, and a partition file was

generated using anvi-get-sequences-for-hmm-hits[40]. A maximum likelihood tree was calculated with FastTree[59] v2.1.11 including bootstrapping, and the tree was visualized in iToL[60] v6.8.1. Presence of *nifH* and *amt* genes for all *Hyphomicrobiaceae* genomes in the tree (Extended Data Fig. 3) was assessed based on KEGG enzyme annotations. For this, *Hyphomicrobiaceae* genomes were loaded into anvi'o[40] v8, genes called by prodigal[78] v2.6.3, and KEGG[102] functional annotation obtained using HMMER v3.3.2 (http://hmmer.org/).

To calculate the maximum likelihood tree of NifH proteins, NifH amino acid sequences from the 85,205 GTDB species representatives[103] (version r214) were identified using an alignment score ratio approach as previously described[104]. In brief, the protein complement of the 85,205 genomes was used as query in a DIAMOND[50] v2.0.8 search against a self-curated database of NifH sequences (curated from Méheust et al.[105] by removing long sequences). Subsequently, the maximum alignment score of all sequences that had a hit was calculated. To identify the NifH sequences, the ratio of alignment score against the database and maximum alignment score was used. NifH sequences were then aligned using muscle[58] v3.8.1551 and a preliminary phylogenetic tree was calculated using FastTree[59] v2.1.11 with default parameters. This preliminary phylogenetic tree was used to discern group I NifH proteins from those that belong to other groups[105]. For the final phylogenetic analysis, group I NifH sequences obtained from the Pseudomonadota, the *Ca*. T. diatomicola NifH from the Marine Atlas of Tara Ocean Unigenes (MATOU-v1_23614344)[97], the NifH from the *Ca*. T. diatomicola and *Ca.* T. profundi genomes as well as the cyanobacterial NifH sequences from the Méheust et al.[105] reference dataset (outgroup) were used. The selected sequences were aligned using MAFFT[72] v7.505 ("--auto"). The alignment was trimmed using trimAl[73] v1.4.1. A maximum likelihood phylogenetic tree was calculated using IQ-TREE[76] v2.2.0.3 with automated model selection using ModelFinder[74] including 1000 ultrafast bootstraps using UFBoot2 (ref. [75]). The resulting tree was visualized in iTOL[60] v6.8.1. For NifDKENBS, phylogenetic trees were created the same way as for NifH, using proteins from the same Pseudomonadota genomes included in the NifH

phylogeny. The NifDKENBS proteins were identified in these genomes based on homology to the respective *Ca.* T. diatomicola proteins using the alignment score ratio approach described above. Because NifEN are encoded as one large fusion protein in some genomes (both *Ca.* Tectiglobus genomes, the closest *nifH* relative and some cyanobacterial genomes), they were manually separated into the NifE and NifN domains based on domain annotation using the InterPro web server[54] v98.0 (https://www.ebi.ac.uk/interpro/result/InterProScan). These separated NifE and NifN domains were then used for creating the phylogenetic trees.

To calculate the maximum likelihood tree of CcoN proteins, CcoN amino acid sequences were identified and retrieved from the protein complement of the 85,205 GTDB species representatives[103] (version r214), using a curated reference database of CcoN sequences from Murali et al.[106]. The identified CcoN sequences were clustered at 70% sequence identity using USEARCH[71] v11.0.667 and the *Ca*. T. diatomicola and *Ca.* T. profundi CcoN sequences were added to the dataset. The clustered sequence set was aligned using muscle[58] v3.8.1551 and a preliminary phylogenetic tree was calculated using Fasttree[59] v2.1.11 followed by selection of sequences representing the broad phylogenetic neighborhood of the *Ca*. T. diatomicola CcoN. A phylogenetic tree of these selected sequences was calculated the same way as the preliminary phylogenetic tree. The 92 sequences representing the close phylogenetic neighborhood of the *Ca*. T. diatomicola CcoN sequence were then selected for the final CcoN phylogenetic analysis. The final CcoN phylogenetic tree was calculated with IQ-TREE[76] v2.2.2.7, using the LG+F+I+G4 model after evaluation with ModelFinder[74] including 1000 ultrafast bootstraps using UFBoot2[75]. The resulting tree was visualized in iTOL[60] v6.8.1.

Bulk rates of CO_2 and N_2 fixation

Rates of CO_2 and N_2 fixation were determined following the approach of Großkopf et al.[85] and Martínez-Pérez et al.[10]. Stable isotope incubations were carried out in triplicates in 4.7-

L transparent polycarbonate bottles (Nalgene; cleaned with hydrochloric acid prior to the cruise). These were amended with ^{13}C-bicarbonate ($NaH^{13}CO_3$; ≥ 98 at% ^{13}C; Sigma-Aldrich) and ^{15}N-N_2 (Cambridge Isotope Laboratories, obtained via Eurisotop; lot numbers: I-21065/AR0664758 (MSM89), I-19197/AR0586172 and I-21065/AR0664758 (M161)) to reach enrichment levels of 5.7-6.8 (Mean= 6.1, SD= 0.3; both cruises combined) at% for ^{13}C in the dissolved inorganic carbon (DIC) pool and 9.1-14.0 (Mean= 11.5, SD= 1.6; MSM89 cruise) and 4.5-8.1 (Mean= 6.4, SD= 1.1; M161 cruise) at% for ^{15}N in the N_2 pool. The ^{15}N-N_2 gas was checked for ^{15}N-NH_4^+ contamination using the hypobromite method[107] prior to the cruises, and no contamination was detected. Heavy stable isotope enrichment of each substrate pool was determined from subsamples taken at the end of the incubations from every isotope-amended bottle using membrane inlet mass spectrometry (for ^{15}N in the N_2 pool) and cavity ring-down spectroscopy (for ^{13}C in the DIC pool). The $^{15}N_2$ gas was added using a modified bubble method[108,109] for which the bottles were gently agitated for 15-20 min prior to bubble removal. Bottles were incubated headspace-free in on-deck incubators, which were continuously flushed with surface seawater under simulated ambient light conditions[86] (36% light transmission, Lee filter, no. 724, 'Ocean Blue' for surface waters). After ~ 24 h, 3.5 L of incubated seawater were filtered onto pre-combusted (4-6 h at 450 °C) GF/F filters (Whatman; diameter 25 mm) for bulk elemental and isotopic analyses, and 100-120 mL were preserved for FISH and nanoSIMS analyses (see below). GF/F filters were dried (55-65 °C overnight) on board and/or frozen at -20 °C until further processing. The elemental and isotopic composition of the collected biomass was determined using an elemental analyzer (Thermo Flash EA, 1112 Series) coupled to a continuous-flow isotope ratio mass spectrometer (Delta Plus XP IRMS; Thermo Fisher Scientific) after GF/F filters were decalcified overnight in an acidic atmosphere (using fuming hydrochloric acid). CO_2 and N_2 fixation rates were subsequently calculated based on the incorporation of ^{13}C and ^{15}N into biomass (i.e. the change in isotopic composition) according to Großkopf et al.[85] and Martínez-Pérez et al.[10]. Natural abundances for the calculations were

obtained from unamended single control bottles incubated alongside the isotope-amended bottles. These samples were processed the same way as described above. All rates are reported as averages of triplicates. Samples for FISH (from the start of incubations and depth profiles) and nanoSIMS were fixed using methanol-free paraformaldehyde solution (1% w/v final concentration) either for ~ 24 h at 4 °C or for a few hours at 4 °C followed by 0.5 h at room temperature. Fixed FISH and nanoSIMS samples were subsequently filtered onto polycarbonate and gold (Au)-coated polycarbonate filters (Isopore, 0.2 µm pore size, 25 mm diameter), respectively. All filters were subsequently rinsed with ultrapure water (MilliQ) before being dried and stored at -20 °C until further analyses.

Design and optimization of FISH-probes targeting *Ca.* T. diatomicola

For fluorescence *in situ* hybridization (FISH) probe design, the *Ca.* T. diatomicola 16S rRNA sequence obtained in this study was imported into ARB[110] (version arb-devel-7.1.rev19270) and aligned to the SILVA_138.1_SSURef_NR99_12_06_20 database[111] using the SINA aligner integrated into ARB[110]. FISH probes were designed using the probe design tool in ARB and evaluated in silico using MathFISH[112]. Unlabeled competitor probes were designed for non-target organisms, if the difference between the in silico-predicted formamide melting concentrations of target and non-target organisms was less than 20% (ref. [112]). In addition, for probes that bind to regions of the 16S rRNA with low accessibility[113], unlabeled helper probes (with equal or higher in silico predicted formamide melting concentration) were designed to enhance probe binding to the target organisms. The newly designed probes were tested and optimized using Clone-FISH[89]. For this, paraformaldehyde-fixed *E. coli* cells (JM109(DE3)) containing the full-length 16S rRNA gene sequence of *Ca*. T. diatomicola on a peT-23a(+) plasmid (obtained from GenScript) were prepared as previously described[89]. Formamide melting curves[114] were performed using the newly designed probes in the form of double-Atto488-

labelled probes (DOPE-FISH[115]), together with all corresponding unlabeled competitor and/or helper probes[116]. Fixed *E. coli* cells were applied to Teflon-coated glass microscope slides (Marienfeld, Germany), dried and dehydrated using increasing concentrations of ethanol. FISH was performed using 0, 5, 10, 15, 20, 25, 30, 35, 40, 45, 50, 60 and 70% formamide in the hybridization buffer, as previously described[116]. For each formamide concentration, at least four images were acquired on an Axio Imager 2 microscope (Zeiss) of random fields of view using identical exposure settings for all formamide concentrations. Formamide melting curves were analyzed using daime[117] v2.2.2 (see Extended Data Table 2 for optimal formamide concentrations).

Scanning electron microscopy (SEM)

To identify and characterize the host of *Ca*. T. diatomicola, diatoms containing *Ca*. T. diatomicola cells were visualized by scanning electron microscopy (SEM) with a FEI Quanta 250 FEG ESEM (Thermo Fisher Scientific, FEI). Secondary electron micrographs were taken using a primary electron beam with an acceleration voltage of 2 kV and an Everhart-Thornley detector for the detection of the secondary electrons on Au-coated filters. For non-coated filters, the low vacuum mode of the SEM was used for imaging with a pressure of around $9e^{-1}$ mbar and an acceleration voltage of 5 kV for the primary electrons. The secondary electrons micrographs were taken with a Large Field Gaseous (LFD) detector.

Single-cell activities using nanoSIMS

Single-cell C and N isotopic composition of *Ca*. T. diatomicola and its *Haslea* hosts as well as *Richelia* and its diatom hosts were determined using nanoscale secondary ion mass spectrometry (nanoSIMS; nanoSIMS50L, CAMECA). Filter areas containing marked diatoms

were pre-sputtered with a Cs^+ ion beam (~300 pA) to remove surface contamination and the outer silica layer of the diatom. Samples were then measured with a Cs^+ ion beam intensity of 1-1.5 pA and a beam diameter of ~100 nm. Each analysis was carried out with a dwell time of 1 ms per pixel and raster sizes of 20 µm x 20 µm to 40 µm x 40 µm, each with 256 pixel x 256 pixel. The secondary ions $^{12}C^-$, $^{13}C^-$, $^{12}C^{14}N^-$, $^{12}C^{15}N^-$, $^{31}P^-$ and $^{32}S^-$ were recorded simultaneously in up to 60 planes for each measurement. Look@nanoSIMS[118] v2018 was used to process the data, including dead-time and drift correction, and accumulating the recorded planes. The isotopic ratios ($^{13}C/^{12}C$ and $^{12}C^{15}N/^{12}C^{14}N$) of regions of interest were determined by overlaying epifluorescence images of the FISH-positive cells and their host diatom (*Ca*. T. diatomicola) or autofluorescence images (diatom-associated *Richelia*) with the nanoSIMS secondary electron images. Only cells with a Poisson error of less than 5% were considered for further analyses. Ratios of $^{15}N/^{14}N$ (from $^{12}C^{15}N/^{12}C^{14}N$) and $^{13}C/^{12}C$ were then used to compute the cell specific N and C assimilation rate of individual cells following the equation below:

$$\text{Cellular rate (C or N cell}^{-1}\text{ d}^{-1}) = (\text{at\%}_{cell} - \text{at\%}_{control}) / (\text{at\%}_{CO2\ or\ N2} - \text{at\%}_{control}) * C_{cell} \text{ or } N_{cell} * 1/t$$

Where $\text{at\%}_{cell}$, $\text{at\%}_{control}$, $\text{at\%}_{CO2\ or\ N2}$ are the atom% ^{13}C or ^{15}N in the individual cell, the natural abundance/control atom% ^{13}C or ^{15}N from the bulk incubation, the atom% ^{13}C or ^{15}N in the DIC or N_2 substrate pools, respectively; C_{cell} or N_{cell} are the C or N biomass per cell, respectively, and t is the incubation time in days. The cellular C biomass was determined from biovolumes and a biovolume-to-biomass conversion. Biovolumes were computed from the size measurements obtained during SEM analysis (for diatom hosts of *Ca*. T. diatomicola; height was assumed as ‘0.6 x width’[119]), nanoSIMS analysis (for *Ca*. T. diatomicola and *Richelia*) or brightfield/epifluorescence microscopy (*Richelia* hosts). The prolate spheroid model and the

prism-on-elliptic base model were used for *Ca*. T. diatomicola and its host, respectively[120]. *Richelia* and their hosts were processed using the prolate spheroid and cylinder+2 cones models[120]. Biovolumes were subsequently converted to C biomass using equations provided by Verity et al.[121] for diatom hosts and *Richelia* and Khachikyan et al.[122] for *Ca*. T. diatomicola, which takes into account the higher C density of smaller cells. Cellular C biomass was converted to cellular N biomass using the Redfield ratio of 6.6:1 for C:N.

In order to determine how much of the fixed C and N remained inside the hosts and symbionts within each *Ca.* T. diatomicola-*Haslea* symbiosis, the total fixation rates were mass-balanced as follows:

$$B_{(total)} = B_{(host)} + A * B_{(symb.)}$$

with B equals biomass as either carbon (C) or nitrogen (N), $B_{(total)}$ in fmol C or N symbiosis^{-1} d^{-1} and $B_{(host)}$ or $B_{(symb)}$ in fmol C or N cell^{-1} d^{-1}, and A as the average number of symbionts per host. Solving for either host or symbiont, the amount of fixed C and N can be expressed as % recovered in either host or symbiont.

Cellular rates and abundances were combined to determine the absolute contributions of the *Ca*. T. diatomicola-*Haslea* symbiosis and diatom-associated *Richelia* to the bulk N_2 fixation rate as follows:

$$Nfix_{group} = Nfix_{cell} * Abundance_{cell}$$

where $Nfix_{group}$, $Nfix_{cell}$, and $Abundance_{cell}$ are the group-specific contribution, the cellular rate (either *Ca*. T. diatomicola-*Haslea* symbiosis or *Richelia* symbioses), and the abundance (of either *Ca*. T. diatomicola-*Haslea* symbiosis or *Richelia* symbioses), respectively. For the *Ca*. T.

diatomicola-*Haslea* symbiosis and the diatom-associated *Richelia*, the amount of N recovered in the diatom hosts was also taken into account.

Supplementary Discussion

Detailed description of diatoms hosting *Ca.* T. diatomicola

Fluorescence and SEM images were used to identify and characterize the diatoms containing *Ca.* T. diatomicola cells. The frustules available for examination were not well preserved with most frustules collapsed into a more or less two-dimensional structure (so that occasionally both external and internal features were visible in the same frustule) (Extended Data Fig. 4). However, they were clearly identified as naviculoid pennate diatoms likely belonging to the genus *Haslea*.

The observed cells varied in length between 19.8 and 57.8 µm (Mean= 32.2, Standard deviation = 12.7, n= 9), with a central transapical axis of between 3.2 and 8.1 µm (Mean= 4.9, Standard deviation = 1.4). They contained two lobed chloroplasts on either side of the central nucleus with the lobes running in parallel to the apical axis (Extended Data Fig. 4f). All cells were lanceolate in shape with acute apices. As described by Round et al.[123], in some specimens the valve apices appeared slightly twisted along the apical axis. Rows of uniseriate transapical striae, were visible, with about 30 transapical stria in 10 µm. This is within a range also described for other *Haslea* species[25]. These structures were overlaid externally by longitudinal strips.

The raphe fissure was bordered internally by ridges. The ridges extended along most of the raphe but terminated just before the valve pole (Extended Data Fig. 4h). The ribs along the raphe were slightly asymmetric in the central valve area (arrow and arrowhead in Extended Data Fig. 4g and j, respectively). These structures were clearly discernable. However, the raphe slit itself and the raphe endings were mostly not seen in the samples (the latter possibly obscured or modified by the symbiont, the former is usually hidden by the bordering ridges). Round et al.[123] have described thickened central costae in epipelic members of *Haslea* but mentioned that in planktonic forms these seem to be absent. As the specimen examined in the present study were all planktonic, the absence of such thickened areas would not contradict their assignment to the genus *Haslea*. *Haslea* species without this characteristic have also been described by other authors (e.g. ref. [25]).

Identification of *Ca.* T. profundi *nif* genes

The closest relative of *Ca*. T. diatomicola, *Ca.* T. profundi, was identified from a metagenome recovered from sediment trap particles collected at station ALOHA in the North Pacific (GCA-013214245) (ref. [99]). Similar to *Ca.* T. diatomicola, the genome of *Ca.* T. profundi was reduced in size and GC content (Extended Data Fig. 3), suggesting a similar obligate symbiotic lifestyle. Unlike in *Ca.* T.diatomicola though, initially no *nif* genes were identified in the genome of *Ca.* T. profundi. Thus, in an effort to recruit *nif* genes potentially missed in the original study, the *Ca.* T. profundi genome (GCA-013214245) was iteratively extended and refined. This led to the recruitment of *nif* genes (Supplementary Table 5) including a *nifH* that is 95% identical to the one of *Ca*. T. diatomicola (Extended Data Fig. 2). An identical *nifH* was also found in a MAG assigned to *Micavibrionaceae* (GCA-013215815), derived from the same sediment trap[99]. Manual inspection of the *Micavibrionaceae* MAG using anvi'o[40] v7.1 revealed that the *nifH*-encoding contig was incorrectly assigned to this MAG. The refined *Ca.* T. profundi

genome further contains a partial 16S rRNA gene sequence that is ~90% similar to the one of *Ca.* T. diatomicola. Our combined results indicate that *Ca.* T. profundi likely also represents an N_2-fixing algae symbiont.

Description of the *Candidate* genus Tectiglobus

Using GTDB-TK[47], *Ca.* T. diatomicola was initially assigned to the undescribed *Hyphomicrobiaceae* genus GCA-2689605, which also comprised the genome of the closest relative *Ca.* T. profundi (GCA-013214245) together with the GTDB genus representative GCA-2689605. However, analysis of the amino acid identities (AAIs) between the genomes (calculated using CompareM) revealed that GCA-2689605 is only 63-64% identical to the two *Ca.* Tectiglobus genomes (which are 72% identical to each other), values at the lower end observed for members of the same genus[124]. Furthermore, GCA-2689605 was more similar to the closest related genome outside of this genus (GCA-905480435) with 65% AAI (to which the two *Ca.* Tectiglobus genomes are only 60-61% identical). These findings, together with the similarities between the two *Ca.* Tectiglobus genomes (Extended Data Fig. 2, 3, 6 and 8) prompted us to place the two *Ca.* Tectiglobus genomes into the novel genus *Ca.* Tectiglobus.

Supplementary References

97 Cornejo-Castillo, F. M. & Zehr, J. P. Intriguing size distribution of the uncultured and globally widespread marine non-cyanobacterial diazotroph Gamma-A. *ISME J* **15**, 124-128, doi:10.1038/s41396-020-00765-1 (2021).

98 Parks, D. H., Imelfort, M., Skennerton, C. T., Hugenholtz, P. & Tyson, G. W. CheckM: assessing the quality of microbial genomes recovered from isolates, single cells, and metagenomes. *Genome Research* **25**, 1043-1055, doi:10.1101/gr.186072.114 (2015).

99 Poff, K. E., Leu, A. O., Eppley, J. M., Karl, D. M. & DeLong, E. F. Microbial dynamics of elevated carbon flux in the open ocean's abyss. *Proceedings of the National Academy of Sciences* **118**, e2018269118, doi:10.1073/pnas.2018269118 (2021).

100 Boeuf, D. *et al.* Biological composition and microbial dynamics of sinking particulate organic matter at abyssal depths in the oligotrophic open ocean. *Proceedings of the*

National Academy of Sciences **116**, 11824-11832, doi:10.1073/pnas.1903080116 (2019).
101 Delmont, T. O. Marine diazotrophs. doi:https://doi.org/10.6084/m9.figshare.14248283.v2 (2021).
102 Kanehisa, M., Furumichi, M., Tanabe, M., Sato, Y. & Morishima, K. KEGG: new perspectives on genomes, pathways, diseases and drugs. *Nucleic Acids Research* **45**, D353-D361, doi:10.1093/nar/gkw1092 (2017).
103 Parks, D. H. *et al.* A complete domain-to-species taxonomy for Bacteria and Archaea. *Nat Biotechnol* **38**, 1079-1086, doi:10.1038/s41587-020-0501-8 (2020).
104 Speth, D. R. & Orphan, V. J. Metabolic marker gene mining provides insight in global mcrA diversity and, coupled with targeted genome reconstruction, sheds further light on metabolic potential of the Methanomassiliicoccales. *PeerJ* **6**, e5614, doi:10.7717/peerj.5614 (2018).
105 Méheust, R. *et al.* Groundwater Elusimicrobia are metabolically diverse compared to gut microbiome Elusimicrobia and some have a novel nitrogenase paralog. *ISME J* **14**, 2907-2922, doi:10.1038/s41396-020-0716-1 (2020).
106 Murali, R., Hemp, J., Orphan, V. & Bisk, Y.FIND: Identifying functionally and structurally important features in protein sequences with deep neural networks. *bioRxiv* (2019): 592808.
107 Warembourg, F. R. in *Nitrogen Isotope Techniques* (eds Roger Knowles & T. Henry Blackburn) 127-156 (Academic Press, 1993).
108 Klawonn, I. *et al.* Simple approach for the preparation of $^{15-15}N_2$-enriched water for nitrogen fixation assessments: evaluation, application and recommendations. *Frontiers in Microbiology* **6** (2015).
109 White, A. E. *et al.* A critical review of the $^{15}N_2$ tracer method to measure diazotrophic production in pelagic ecosystems. *Limnology and Oceanography: Methods* **18**, 129-147, doi:10.1002/lom3.10353 (2020).
110 Ludwig, W. *et al.* ARB: a software environment for sequence data. *Nucleic Acids Research* **32**, 1363-1371, doi:10.1093/nar/gkh293 (2004).
111 Yilmaz, P. *et al.* The SILVA and "All-species Living Tree Project (LTP)" taxonomic frameworks. *Nucleic Acids Research* **42**, D643-D648, doi:10.1093/nar/gkt1209 (2014).
112 Yilmaz, L. S., Parnerkar, S. & Noguera, D. R. mathFISH, a Web Tool That Uses Thermodynamics-Based Mathematical Models for In Silico Evaluation of Oligonucleotide Probes for Fluorescence In Situ Hybridization. *Applied and Environmental Microbiology* **77**, 1118-1122, doi:10.1128/AEM.01733-10 (2011).
113 Fuchs, B. M., Glöckner, F. O., Wulf, J. & Amann, R. Unlabeled Helper Oligonucleotides Increase the In Situ Accessibility to 16S rRNA of Fluorescently Labeled Oligonucleotide Probes. *Applied and Environmental Microbiology* **66**, 3603-3607, doi:10.1128/AEM.66.8.3603-3607.2000 (2000).
114 Manz, W., Amann, R., Ludwig, W., Wagner, M. & Schleifer, K.-H. Phylogenetic Oligodeoxynucleotide Probes for the Major Subclasses of Proteobacteria: Problems and Solutions. *Systematic and Applied Microbiology* **15**, 593-600, doi:10.1016/S0723-2020(11)80121-9 (1992).
115 Stoecker, K., Dorninger, C., Daims, H. & Wagner, M. Double Labeling of Oligonucleotide Probes for Fluorescence In Situ Hybridization (DOPE-FISH) Improves Signal Intensity and Increases rRNA Accessibility. *Applied and Environmental Microbiology* **76**, 922-926, doi:10.1128/AEM.02456-09 (2010).
116 Daims, H. Use of fluorescence in situ hybridization and the daime image analysis program for the cultivation-independent quantification of microorganisms in environmental and medical samples. *Cold Spring Harb. Protoc* **4**, 1-8 (2009).

117 Daims, H., Lücker, S. & Wagner, M. daime, a novel image analysis program for microbial ecology and biofilm research. *Environmental Microbiology* **8**, 200-213, doi:10.1111/j.1462-2920.2005.00880.x (2006).
118 Polerecky, L. *et al.* Look@NanoSIMS – a tool for the analysis of nanoSIMS data in environmental microbiology. *Environmental Microbiology* **14**, 1009-1023, doi:10.1111/j.1462-2920.2011.02681.x (2012).
119 Olenina, I. Biovolumes and size-classes of phytoplankton in the Baltic Sea. (2006).
120 Sun, J. & Liu, D. Geometric models for calculating cell biovolume and surface area for phytoplankton. *Journal of Plankton Research* **25**, 1331-1346, doi:10.1093/plankt/fbg096 (2003).
121 Verity, P. G. *et al.* Relationships between cell volume and the carbon and nitrogen content of marine photosynthetic nanoplankton. *Limnology and Oceanography* **37**, 1434-1446, doi:10.4319/lo.1992.37.7.1434 (1992).
122 Khachikyan, A. *et al.* Direct Cell Mass Measurements Expand the Role of Small Microorganisms in Nature. *Applied and Environmental Microbiology* **85**, e00493-00419, doi:10.1128/AEM.00493-19 (2019).
123 Round, F. E., Crawford, R. M. & Mann, D. G. *Diatoms: Biology and Morphology of the Genera*. (Cambridge University Press, 1990).
124 Konstantinidis, K. T. & Tiedje, J. M. Towards a Genome-Based Taxonomy for Prokaryotes. *Journal of Bacteriology* **187**, 6258-6264, doi:10.1128/jb.187.18.6258-6264.2005 (2005).
