# Supplementary material for: Rhizobia–diatom symbiosis fixes missing nitrogen in the ocean
Source: Nature. 2024 May 9;630(8018):899–904. doi: 10.1038/s41586-024-07495-w (PMC11208148; doi:10.1038/s41586-024-07495-w)
Supplement: Supplementary file 2 — Reporting Summary [file 41586_2024_7495_MOESM2_ESM.pdf]

nature portfolio

Corresponding author(s): Marcel Kuypers

Last updated by author(s): Apr 17, 2024

Reporting Summary

Nature Portfolio wishes to improve the reproducibility of the work that we publish. This form provides structure for consistency and transparency in reporting. For further information on Nature Portfolio policies, see our Editorial Policies and the Editorial Policy Checklist.

Statistics

For all statistical analyses, confirm that the following items are present in the figure legend, table legend, main text, or Methods section.

n/a	Confirmed	
☐	☒	The exact sample size (n) for each experimental group/condition, given as a discrete number and unit of measurement
☐	☒	A statement on whether measurements were taken from distinct samples or whether the same sample was measured repeatedly
☒	☐	The statistical test(s) used AND whether they are one- or two-sided<br>*Only common tests should be described solely by name; describe more complex techniques in the Methods section.*
☒	☐	A description of all covariates tested
☒	☐	A description of any assumptions or corrections, such as tests of normality and adjustment for multiple comparisons
☐	☒	A full description of the statistical parameters including central tendency (e.g. means) or other basic estimates (e.g. regression coefficient) AND variation (e.g. standard deviation) or associated estimates of uncertainty (e.g. confidence intervals)
☒	☐	For null hypothesis testing, the test statistic (e.g. F, t, r) with confidence intervals, effect sizes, degrees of freedom and P value noted<br>*Give P values as exact values whenever suitable.*
☒	☐	For Bayesian analysis, information on the choice of priors and Markov chain Monte Carlo settings
☒	☐	For hierarchical and complex designs, identification of the appropriate level for tests and full reporting of outcomes
☒	☐	Estimates of effect sizes (e.g. Cohen's d, Pearson's r), indicating how they were calculated

Our web collection on statistics for biologists contains articles on many of the points above.

Software and code

Policy information about availability of computer code

Data collection	Zeiss ZEN 3.2 blue edition; for the acquisition of raw mass spectrometric data, instrument-supplied software was utilized; also see Methods and Supplementary Information.
Data analysis	Trimmomatic v0.39, MEGAHIT v1.2.9, anvi'o v7.1 and v8, BLAST+ v2.9.0, minimap2 v2.22-r1101, SAMtools v1.14, CoverM v0.6.1, seqkit v2.3.0, SPAdes v3.15.3, CheckM2 v0.1.2, GTDB-TK v2.1.0, Prokka v1.14.6, DIAMOND v2.0.8, SqueezeMeta v1.6.2, mauve development snapshot 2015-02-26, muscle v3.8.1551, IQ-TREE v2.2.0.3 and v2.2.2.7, iToL v6.8.1, BWA-MEM v0.7.17-r1188, featureCounts v2.0.1, BRIG v0.95, DNAPlotter v18.1.0, bbmap v38.70/v38.87/v38.98, R v4.0.2, hifiasm-meta v0.2-r043, CompareM v0.1.2, fastANI v1.33, USEARCH v11.0.667, FastTree v2.1.11, MAFFT v7.505, trimAl v1.4.1, Barrnap 0.9-dev, HMMER v3.1b2 and v3.3.2, prodigal v2.6.3, Bowtie2 v2.3.4.1, MetaBAT 2 v2.12.1, MaxBin 2.0, CONCOCT v1.1.0, DAS Tool v1.1.1, ARB version arb-devel-7.1.rev19270, daime v2.2.2, Look@nanoSIMS v2018, UFBoot2, ModelFinder, RDP classifier v2.10.2, web servers: RAST, NCBI BLAST, MathFISH (no version numbers available), InterPro v95.0-98.0.<br>Custom scripts for iterative bin refinements are deposited at https://github.com/bresyd/mag_refinement/tree/main

For manuscripts utilizing custom algorithms or software that are central to the research but not yet described in published literature, software must be made available to editors and reviewers. We strongly encourage code deposition in a community repository (e.g. GitHub). See the Nature Portfolio guidelines for submitting code & software for further information.

Data

Policy information about availability of data

All manuscripts must include a data availability statement. This statement should provide the following information, where applicable:

- Accession codes, unique identifiers, or web links for publicly available datasets
- A description of any restrictions on data availability
- For clinical datasets or third party data, please ensure that the statement adheres to our policy

Read data from metagenomic analyses pertaining to Ca. T. diatomicola have been deposited at the NCBI under BioProject accession number PRJNA1036431, including the MAGs of Ca. T. diatomicola and Ca. T. profundi under the accession numbers JAZDSJ000000000 and DAWWJP000000000, respectively. RNA sequencing data can be found under the same bioproject number with the accession numbers SRR26695118, SRR26695119 and SRR26695121- SRR26695130. The publicly available sequences used for phylogenetic tree construction and genome comparison can be found at the Genome Taxonomy Database (GTDB; https://gtdb.ecogenomic.org/) under the accession numbers given in Supplementary Data 1-9 (tree file for each tree). Publicly available MAGs from Delmont et al. (2022) can be found under https://figshare.com/articles/dataset/Marine_diazotrophs/14248283. Tara ocean metagenomes used in this study can be found under https://www.ncbi.nlm.nih.gov/bioproject/173486 with the following bioprojects used in this study: PRJEB4352 (size fractions for protists; https://www.ncbi.nlm.nih.gov/bioproject/213098), PRJEB1787 (size fractions for prokaryotes; https://www.ncbi.nlm.nih.gov/bioproject/196960), PRJEB9691 (size fractions for protists from polar circle samples; https://www.ncbi.nlm.nih.gov/bioproject/287904), PRJEB9740 (size fractions for prokaryotes from polar circle samples; https://www.ncbi.nlm.nih.gov/bioproject/288558).
For the reconstruction of the MAG of Ca. T. profundi, the used metagenomic data can be found at the GTDB under: https://gtdb.ecogenomic.org/genome?gid=GCA_013214245.1 (original MAG) as well as the Sequence Read Archive under: https://www.ncbi.nlm.nih.gov/bioproject/PRJNA482655 with the accession numbers SRR7648332, SRR7648341, SRR7648350, SRR7632647, SRR7648334 (metagenomes used for MAG reconstruction). Publicly available qPCR data from Shao et al. (2023) can be found under: https://doi.org/10.6084/m9.figshare.21677687.v3. Additional databases used in this study can found under the following links: eggNOG: http://eggnog45.embl.de/download/eggnog_4.5/data/NOG/, ncbi-nr database: https://ftp.ncbi.nlm.nih.gov/blast/db/FASTA/nr.gz, pfam database: https://www.ebi.ac.uk/interpro/download/pfam/, kegg database: http://andes.cnb.csic.es/SqueezeMeta/kegg.db.gz. Source data are provided with this paper.

Research involving human participants, their data, or biological material

Policy information about studies with human participants or human data. See also policy information about sex, gender (identity/presentation), and sexual orientation and race, ethnicity and racism.

Reporting on sex and gender	N/A
Reporting on race, ethnicity, or other socially relevant groupings	N/A
Population characteristics	N/A
Recruitment	N/A
Ethics oversight	N/A

Note that full information on the approval of the study protocol must also be provided in the manuscript.

Field-specific reporting

Please select the one below that is the best fit for your research. If you are not sure, read the appropriate sections before making your selection.

☐ Life sciences ☐ Behavioural & social sciences ☒ Ecological, evolutionary & environmental sciences

For a reference copy of the document with all sections, see nature.com/documents/nr-reporting-summary-flat.pdf

Ecological, evolutionary & environmental sciences study design

All studies must disclose on these points even when the disclosure is negative.

Study description	The study is based on two parallel cruises (R/V MARIA S. MERIAN (cruise MSM89; Bridgetown, Barbados – Bridgetown, Barbados) and R/V METEOR (cruise M161; Bridgetown, Barbados – Ponta Delgada, Azores, Portugal) in January/February 2020 in the western tropical North Atlantic. The aim of our participation on these cruises was to determine nitrogen fixation rates and identify N2 fixers present in this region. For this, on-deck experiments were carried out to determine in-situ N and C fixation rates in surface water samples (with incubations going for 24 hrs from dawn to dawn). At the same time, microbial biomass from surface waters was sampled using filtration for subsequent DNA/RNA extractions and sequencing, to identify potential novel N2-fixers and study their metabolic potential. Samples were also for visualization of specific microorganims using FISH and determination of single cell rates using nanoSIMS.
Research sample	The tropical North Atlantic is an area known to harbor an active community of N2-fixers (accounting for ~20% of the oceanic N2

Research sample	fixation). It was also known that typical N2 fixers can not account for the bulk rates previously measured in this area. The combination of high N2 fixation and the potential presence of so far unknown N2-fixers was the crucial factor for our decision to chose this study site. In detail, we report results from sequencing data and/or incubation experiments from eight different surface water stations (six from the MSM89 and two from the M161 cruise).
Sampling strategy	No statistical methods were used to pre-determine sample size. Sampling size was largely determined by the feasibility of experiments with incubation experiments carried out in triplicates for each station. Please also refer to the methods section.
Data collection	Surface water for all experiments and samples was collected using rosette samplers equipped with CTD (conductivity-temperature-depth) systems and Niskin bottles by the CTD team on each cruise. Biomass and mass spectrometric data was collected using an elemental analyzer coupled to a continuous-flow isotope ratio mass spectrometer (equipped with an autosampler, operated by technical support staff), a nanoscale secondary ion mass spectrometer (operated by Abiel Kidane and technical support staff), a gas chromatograph and a gas chromatograph coupled to an isotope ratio mass spectrometer (operated by technical support staff). Sequencing data was generated at the Max Planck Genome Centre Cologne (individual sequencing platforms are detailed in the methods section).
Timing and spatial scale	The two parallel cruises took place during January/February 2020. This study reports data from a total of eight stations within an area from 13.7720 - 10.769 °N and 57.7815 - 57.2454 °W that were visited during that time frame.
Data exclusions	No data were excluded from analyses.
Reproducibility	All experiments described in our study come from come from two parallel cruises visiting distinct locations at different days. Within each set of experiments, triplicate incubations and multiple measurements within each triplicate (where applicable) were performed to assess variability. Variability within triplicates was substantial (see Extended Data Table 1) reflecting biological differences between individual microbial communities and in-situ conditions rather than errors in measurements. All attempts at replication were successful.
Randomization	The location for sampling was selected randomly depending on the position of the vessels at dawn on the days of the stations.
Blinding	Blinding was not pertinent to our study because it did not include any animals and/or human research participants. In addition, blinding was not possible since many analyses were also carried out by the persons in charge of sampling and interpretation of the data was done by persons in charge of analyses.

Did the study involve field work? ☒ Yes ☐ No

Field work, collection and transport

Field conditions	Overall, the weather conditions during the two parallel cruises were calm with only some intermittent light rain. The surface water temperature at the stations was ~27 °C.
Location	The dates and locations of the eight stations are as follows: MSM89-S1 (1/19/2020; 13.7720 °N 57.2483 °W), MSM89-S2 (1/21/2020; 12.7599 °N 57.6089 °W), MSM89-S4 (1/25/2020; 11.8008 °N 57.2755 °W), MSM89-S10 (2/7/2020; 12.4191 °N 57.2454 °W), MSM89-S13 (2/14/2020; 10.769 °N 57.7815 °W), MSM89-S14 (2/16/2020; 11.0519 °N 57.2798 °W), M161-S4 (1/27/2020; 12.125 °N 57.2456 °W), M161-S7 (2/4/2020; 12.5879 °N 57.6069 °W).
Access & import/export	Samples were collected and exported in compliance with local, national and international regulations. Permissions to conduct marine scientific research in territorial waters were issued by the Ministry of Foreign Affairs and Foreign Trade in Barbados (No. IR/2020/08 and IR/2020/09; issued January 15th, 2020) and the Ministry of Foreign and CARICOM Affairs of the Republic of Trinidad and Tobago (No. 2053; issued November 12th, 2019).
Disturbance	No disturbances were caused by this study.

Reporting for specific materials, systems and methods

We require information from authors about some types of materials, experimental systems and methods used in many studies. Here, indicate whether each material, system or method listed is relevant to your study. If you are not sure if a list item applies to your research, read the appropriate section before selecting a response.

Materials & experimental systems

n/a	Involved in the study	
☒	☐	Antibodies
☒	☐	Eukaryotic cell lines
☒	☐	Palaeontology and archaeology
☒	☐	Animals and other organisms
☒	☐	Clinical data
☒	☐	Dual use research of concern
☒	☐	Plants

Methods

n/a	Involved in the study	
☒	☐	ChIP-seq
☒	☐	Flow cytometry
☒	☐	MRI-based neuroimaging

Plants

Seed stocks	N/A
Novel plant genotypes	N/A
Authentication	NA
