# Supplementary material for: Rhizobia–diatom symbiosis fixes missing nitrogen in the ocean
Source: Nature. 2024 May 9;630(8018):899–904. doi: 10.1038/s41586-024-07495-w (PMC11208148; doi:10.1038/s41586-024-07495-w)
Supplement: Supplementary file 8 — These are tree files corresponding to the phylogenetic trees shown in Fig.1 and Extended Data Figs. 2 and 3. [file 41586_2024_7495_MOESM8_ESM.zip › 2023-11-20804B-s8/2023-11-20804B-Kuypers_Tree_file_guide.docx]

**Supplementary Data**

Tree files nifH, nifD, nifK, nifE, nifB, nifN and nifS correspond to Extended Data Figure 2b.

Tree file ccoN corresponds to Extended Data Figure 6.

Tree file Rhizobiales corresponds to Figure 1a and Extended Data Figure 3.
